# Differential atrophy along the longitudinal axis of the hippocampus in Alzheimer’s disease and suspected non-Alzheimer’s disease pathophysiology (SNAP)

**DOI:** 10.1007/s00234-026-04061-1

**Published:** 2026-06-06

**Authors:** Torcato Meira, Rafaela Morais-Ribeiro, Tiago Jesus, Marcelo Dias, Ana Coelho, Tiago Gil Oliveira

**Affiliations:** 1 Life and Health Sciences Research Institute (ICVS), School of Medicine, University of Minho, Braga, Portugal; 2 Department of Neuroradiology, ULS / Hospital de Braga, Braga, Portugal

**Keywords:** Alzheimer's disease continuum, Suspected non-Alzheimer's pathophysiology, SNAP, Hippocampus, Hippocampal longitudinal axis, Hippocampal atrophy, Cerebrospinal fluid biomarkers, Amyloid-beta, Tau, Magnetic resonance imaging

## Abstract

**Purpose:**

Cerebrospinal fluid (CSF) biomarkers are increasingly used to support Alzheimer’s disease (AD) diagnosis: Alzheimer’s Disease continuum (ADc) is defined by abnormal amyloid-beta (Aβ), while Suspected Non-AD Pathophysiology (SNAP) refers to normal Aβ with elevated tau. Given the differential properties along the hippocampal longitudinal axis, this study aimed to evaluate anterior-posterior subregional hippocampal volume differences among ADc, SNAP, and biomarker-negative participants, and their associations with CSF biomarkers and clinical presentation.

**Methods:**

Data from 1242 participants in the Alzheimer’s Disease Neuroimaging Initiative were analyzed. Biomarker-negative participants (*n* = 234) had normal CSF Aβ42 (≥ 192 pg/ml), total tau (< 93 pg/ml), and phosphorylated tau at threonine 181 (< 23 pg/ml). ADc individuals (*n* = 784) had abnormal Aβ42, and SNAP (*n* = 224) showed normal Aβ42 with elevated tau. T1-weighted MRI scans were used to segment the hippocampus into anterior, intermediate, and posterior subregions. Controlling for age, sex, and multiple comparisons, groups were compared using one-way ANOVA, and Pearson correlation coefficients were calculated to assess the relationships between volumetric variables and age, CSF biomarkers, or neuropsychological scores.

**Results:**

ADc individuals showed significantly lower total and subregional hippocampal volumes compared with SNAP and biomarker-negative participants (*P* < 0.001). Normalized volumes to total ipsilateral hippocampal volume revealed greater posterior atrophy (*P* < 0.001) and relatively higher anterior volume (*P* < 0.01) in ADc. SNAP and biomarker-negative participants showed stronger correlations between hippocampal volumes and age. Several subregional hippocampal volumes correlated with CSF biomarkers, cognitive performance, neuropsychiatric symptoms, and functional impairment.

**Conclusion:**

This study reveals distinct patterns of hippocampal atrophy in ADc and SNAP, enhancing our understanding of their differential pathophysiology and associated clinical features.

**Supplementary Information:**

The online version contains supplementary material available at https://doi.org/10.1007/s00234-026-04061-1.

## Introduction

Alzheimer’s disease (AD) is characterized by the accumulation of amyloid-β (Aβ) and tau proteins in the brain, leading to neurodegeneration and cognitive decline [1, 2]. Cerebrospinal fluid (CSF) biomarkers are increasingly used in diagnosing neurodegenerative conditions. Biomarker-based criteria were proposed to classify individuals based on their Aβ, tau, and/or neurodegeneration/neuronal injury status [3]. According to these criteria, indirect evidence of aggregated Aβ pathology, such as decreased Aβ42 levels in CSF, defines the Alzheimer’s disease continuum (ADc). The introduction of these criteria has led to the concept of suspected non-AD pathophysiology (SNAP), which describes individuals with no evidence of Aβ pathology but with signs of tau pathology or neurodegeneration, indicated by elevated levels of CSF phosphorylated tau or total tau (t-tau), respectively. In the revised criteria for AD diagnosis and staging, greater emphasis is placed on the biological definition of AD, with Aβ pathology remaining central for its classification [4]. These criteria also stress the need for caution when interpreting abnormal biomarkers in isolation, particularly tau-based measures in the absence of concomitant Aβ pathology, as such profiles may reflect non-AD processes. In this context, elevated CSF tau in Aβ-negative individuals has been linked to neuropathologically defined primary age-related tauopathy (PART) [4], a non-AD condition that remains comparatively understudied but is known to be associated with distinct clinical and imaging features [5, 6]. However, this biomarker profile may also reflect other non-AD tauopathies or neurodegenerative processes [7].

Accordingly, SNAP is best regarded as a biomarker-defined category that may encompass a heterogeneous set of underlying pathophysiological processes, distinct from those characterizing the ADc. One of the brain structures affected early on in AD pathogenesis is the hippocampus, which shows a distinct internal organization and morphology along its longitudinal axis, with region-specific differences from anterior to posterior portions in gene expression, electrophysiology, connectivity, and function [8, 9]. Importantly, accumulating evidence suggests that distinct neuropathological processes may relate to different topographical patterns within the medial temporal lobe and along the hippocampal longitudinal axis. In AD-related pathology, posterior hippocampal involvement has been linked to tau-related neurodegeneration, whereas anterior medial temporal and anterior hippocampal involvement has been more strongly associated with transactive response DNA-binding protein 43 (TDP-43) pathology [10, 11]. In parallel, Aβ and tau appear to differentially affect posterior-medial and anterior-temporal networks connected to the hippocampus, respectively [12]. Recent work in clinically defined AD has also shown greater vulnerability of the posterior hippocampus to atrophy [13], whereas other studies suggest that tau pathology may emerge earlier in anterior medial temporal regions, including the anterior hippocampus, before more posterior hippocampal involvement becomes apparent with disease progression [14]. Additionally, co-pathologies such as cerebrovascular disease and Lewy body pathology may further influence regional atrophy patterns [6, 15].

In this study, we use an algorithm to segment the hippocampus into those subregions to assess atrophy along the longitudinal axis of the hippocampus in both ADc and SNAP, as defined by CSF Aβ42, phosphorylated tau at threonine 181 (p-tau181), and t-tau levels, presumably reflecting the underlying neuropathological changes. We also aimed to determine how these patterns of atrophy could help differentiate between these conditions in individuals below 60 years of age. Additionally, we evaluated how various subregional volumetric metrics correlate with CSF biomarkers, clinical presentation and longitudinal cognitive change.

## Materials and methods

### Research design, subjects and variables

The data utilized in this study were obtained from the Alzheimer’s Disease Neuroimaging Initiative (ADNI) database (adni.loni.usc.edu), established in 2003 as a public–private partnership led by principal investigator Michael W. Weiner, MD. The primary aim of ADNI has been to investigate whether various metrics, including those derived from magnetic resonance imaging (MRI), are associated with cognitive dysfunction and the onset of AD. This initiative received approval from the institutional ethical committees of all participating institutions, and written informed consent was obtained from all research participants and, when applicable, from collateral informants. The ADNI database encompasses data from over 60 sites across the USA and Canada. Following approval and granted access to the data, we retrieved ADNI data from https://ida.loni.usc.edu/ across all available cohorts (ADNI-1, ADNI-GO, ADNI-2, and ADNI-3). Individuals were included based on the availability of CSF biomarkers. ADc was defined as CSF Aβ42 < 192 pg/ml; SNAP had Aβ42 levels ≥ 192 pg/ml with t-tau levels ≥ 93 pg/ml or p-tau181 levels ≥ 23 pg/ml. These CSF biomarker cut-off values were defined according to ADNI Biomarker Core recommendations [16] and have been applied in ADNI-based studies [17]. Biomarker-negative participants were defined solely on the basis of CSF biomarkers (Aβ42 ≥ 192 pg/ml, t-tau < 93 pg/ml, and p-tau181 < 23 pg/ml); cognitive status was not used as an inclusion criterion, and this group may therefore include cognitively impaired individuals. Sex, age, volumetric T1-weighted MRI acquisition, scores on the Mini-Mental State Exam (MMSE), Clinical Dementia Rating (CDR), Neuropsychiatric Inventory Q (NPI-Q), Geriatric Depression Scale (GDS), and Functional Activities Questionnaire (FAQ) collected closest to the date of lumbar puncture were obtained for analysis. To assess longitudinal cognitive change, MMSE and CDR scores collected 24 months after baseline (± 3 months), when available, were also retrieved. Analyses were performed using the total scores of these scales. For descriptive contextualization of the SNAP group, clinician-recorded neurologic diagnoses available in ADNI were also retrieved from the diagnosis and symptoms checklist at the study visit closest to lumbar puncture/CSF collection – these variables were examined descriptively only.

### MRI brain imaging analysis and hippocampus segmentation

Volumetric T1-weighted MRI scans were converted to NIfTI format, downloaded, and subjected to further analysis. Structural data were processed using the standard semiautomatic workflow implemented in FreeSurfer toolkit version 6.0 (http://surfer.nmr.mgh.harvard.edu/). This pipeline involves 31 processing steps, including spatial normalization, skull stripping, intensity normalization, gray-white matter tessellation, and segmentation of cortical, subcortical, and white matter regions. This pipeline has been validated against manual segmentations [18] and is considered reliable across sessions, scanner platforms, updates and field strengths [19]. For the purposes of the present study, only left hippocampus, right hippocampus and estimated total intracranial volume (eTIV) were considered. The segmentation of the hippocampus along its longitudinal axis was performed as described by Morais-Ribeiro et al., enabling an unbiased and standardized segmentation of the hippocampus along its longitudinal axis, independent of anatomical landmarks, allowing the assessment of anterior-posterior atrophy gradients that are not directly captured by conventional segmentation pipelines [13]. Briefly, this segmentation method builds upon an algorithm previously described by Lerma-Usabiaga et al. [20] and is publicly available at https://github.com/anacoelho/HippocampusAxisAnalysis.git. Principal component analysis is initially conducted on all points belonging to the hippocampus to derive the longitudinal axis, which is then refined using quadratic Bézier curve. Subsequently, orthogonal planes to the longitudinal axis are defined to perform the sub-segmentation into three hippocampal subdivisions: anterior, intermediate, and posterior. The volume of each subdivision is computed accordingly. All T1-weighted MRI scans included in this study underwent the standard quality control procedures implemented by the ADNI consortium prior to data release. Following automated hippocampal segmentation, all segmentation outputs were visually inspected for segmentation errors and anatomical plausibility. Additionally, extreme volumetric outliers were identified and excluded as described below.

### Statistical analysis

Subregional volumes were normalized by either dividing absolute volume by the absolute total hippocampus volume or by eTIV, as indicated. Residuals of regression models were calculated for volumetric measures and clinical scores to control for potential confounders. Specifically, residuals were adjusted for age and sex for group comparisons of volumetric parameters, volumetric measures correlations with CSF biomarkers, and comparisons of clinical scores between groups; for sex only in analyses assessing volumetric variation over age; and for age, sex, and baseline clinical score in analyses relating hippocampal volumetric measures to longitudinal cognitive change. Longitudinal cognitive change was quantified as the difference between follow-up and baseline MMSE or CDR scores, using assessments obtained 24 months apart (± 3 months). Group comparisons of volumetric parameters, clinical scores, CSF biomarkers (t-tau, p-tau181, Aβ42), and age were conducted using one-way ANOVA followed by Tukey’s multiple comparisons post hoc test – data are presented as box-whisker plots, with the median (center line), 25th to 75th percentiles (box), and minimal and maximal values. Sex distribution between groups was analyzed using the chi-squared test and illustrated using stacked bar charts. For subgroup analyses of participants younger than 60 years of age, sex distribution was compared using Fisher’s exact test, and age distributions were compared using the Mann–Whitney U test. Pearson correlation coefficients (*r*) were calculated to analyze the relationship of volume metrics with age, and comparisons between groups were performed using the Jennrich test with adjustment for multiple comparisons using Bonferroni correction – scatterplots with linear regression trend lines and 95% confidence intervals were generated to visualize the correlation data. Correlation analyses (*r*) between subregional volumetric metrics and either the clinical scores or CSF biomarkers were represented as heat maps, while controlled for multiple corrections using the Benjamini-Hochberg procedure (FDR < 5%). Pearson correlation coefficients (*r*) were additionally used to assess associations between volumetric measures and longitudinal cognitive change, as well as the association between CSF biomarkers and clinical scores, while controlled for multiple comparison using the Benjamini-Hochberg procedure (FDR < 5%). The discrimination magnitude of the anterior/posterior hippocampal volume ratio between ADc and SNAP was assessed by calculating the area under the curve (AUC) of the receiver operating characteristic (ROC). Outliers were excluded via the ROUT method (Q 0.1%). Results were considered significant when *P* < 0.05, except when adjustment for multiple comparisons was conducted (adjusted significance *P* is indicated in corresponding figures). Tests were two-sided. Stata 16.1 was used for calculating residuals, conducting the Jennrich test, performing chi-squared, Fisher’s exact and Mann–Whitney U tests, graphing volumetric variations over age, and generating ROCs. Prism 7 (GraphPad) was utilized for outlier identification, one-way ANOVA, Pearson coefficients calculation, graphing box-whisker plots, stacked bar charts, and correlational heat maps. To maintain readability in the main manuscript, detailed statistical results are provided in Online supplemental data.

## Results

A total of 784 individuals classified as ADc (defined by abnormal Aβ42 concentration in CSF), 224 SNAP participants (exhibiting normal CSF Aβ42 levels but abnormal t-tau or p-tau181), and 234 biomarker-negative participants (displaying normal levels of Aβ42, t-tau, and p-tau181) were included. As anticipated, ADc individuals exhibited reduced Aβ42 levels compared with the other groups (*P* < 0.001), while no differences were observed between biomarker-negative participants and SNAP in Aβ42 levels. Significant differences were observed among the three groups in both t-tau and p-tau181 levels (*P* < 0.001) (Online supplemental data - Fig S1). ADc participants were older compared with SNAP (*P* = 0.002), with no other significant age differences among the groups. There were no differences in sex proportions between the groups (Online supplemental data - Fig S2). According to the available clinician records, 89 SNAP participants were classified as mild cognitive impairment due to AD (one of whom also had parkinsonism symptoms recorded), 3 as mild cognitive impairment due to other etiology (not specified in the records), and 4 as AD. No explicit clinician-recorded diagnoses such as frontotemporal dementia, Huntington’s disease, progressive supranuclear palsy, corticobasal degeneration, vascular dementia, prion-associated dementia, or other non-AD dementias were documented in these specific SNAP cases.

### Total hippocampus volume

**Fig. 1:**
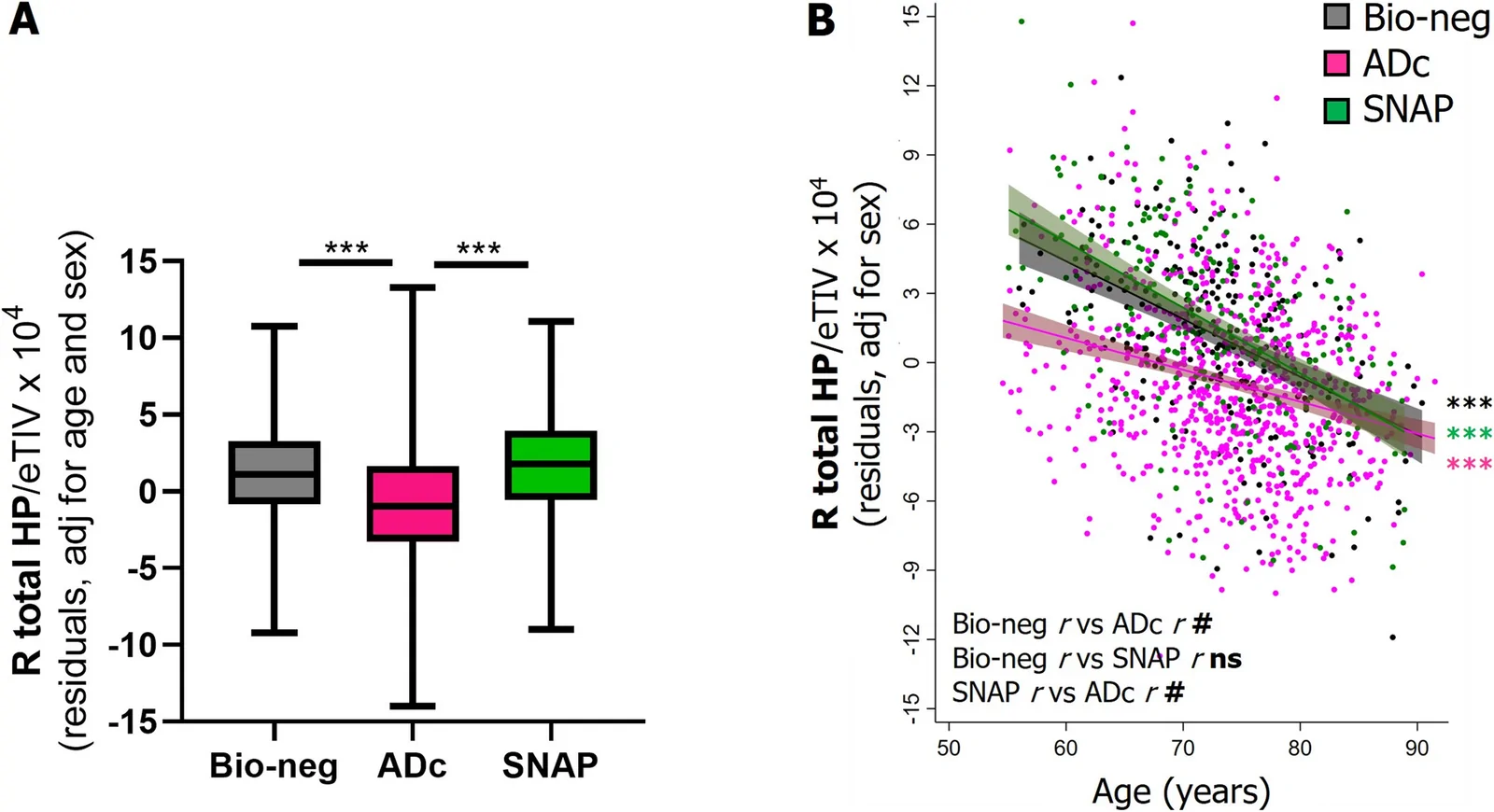
Comparison of total right hippocampus volume between groups. Controlling for age, sex and eTIV, ADc show significantly diminished volumes of the right hippocampus (HP) compared to biomarker-negative participants and SNAP (**A**). Adjusting for sex and eTIV, the three groups show a significant decrease of the right HP volume with age (**B**) - ADc correlation is significantly different from the other groups. Detailed statistical results are provided in Online supplemental data. **#** *P* < 0.01(6); ***P < 0.001; bio-neg, biomarker-negative participants; ns, non-significant; R, right

Initially, we compared the volumes of the total hippocampus among the groups. Controlling for age, sex, and eTIV, ADc participants showed significantly diminished volumes of both right (*P* < 0.001) and left (*P* < 0.001) hippocampus compared with the other groups (Fig. 1A, Online supplemental data – Fig S3A). Adjusting for sex and eTIV, a significant age-related decrease in hippocampal volume was observed bilaterally across all three groups (*P* < 0.001). However, for the right hippocampus, the age–volume correlation in the ADc group was less pronounced compared with biomarker-negative participants (*P* = 0.002) and SNAP (*P* < 0.001); for the left hippocampus, the ADc group showed a significantly different correlation compared with SNAP only (*P* = 0.006) (Fig. 1B, Online supplemental data - Fig S3B).

### Volume of subregions along the longitudinal axis of the hippocampus

**Fig. 2:**
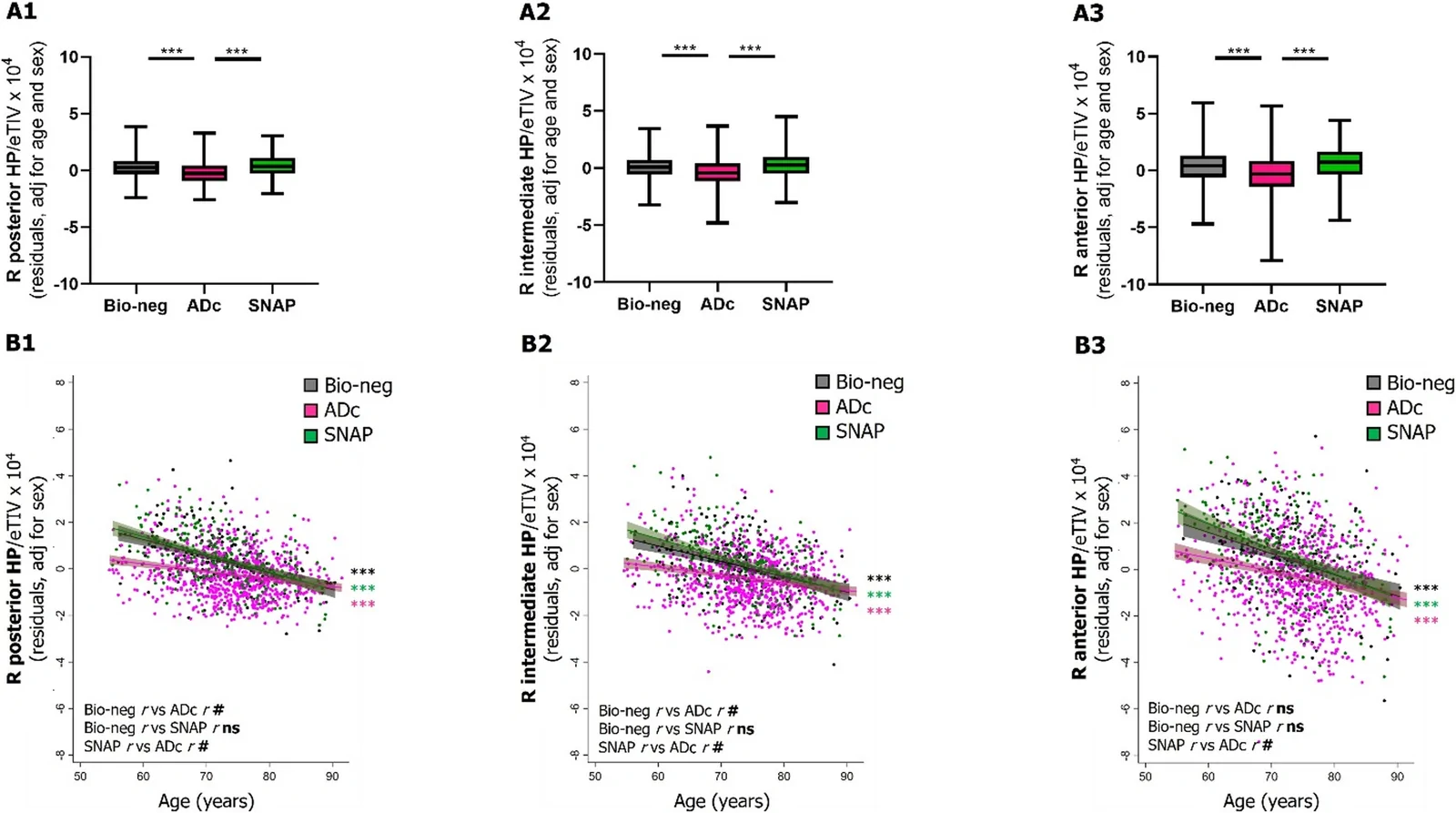
Subregional right hippocampal volumes normalized to eTIV comparisons between groups. On the right hemisphere, ADc displays reduced volume of posterior (**A1**), intermediate (**A2**) and anterior hippocampus (HP) (**A3**). The three groups have a significant decrease of the right posterior (**B1**), intermediate (**B2**) and anterior HP volume (**B3**). ADc correlation with age is significantly different from the remaining groups in posterior HP and in intermediate; in anterior HP, it is different from SNAP. Detailed statistical results are provided in Online supplemental data. **#** *P* < 0.025; ***P < 0.001; bio-neg, biomarker-negative participants; ns, non-significant; R, right

Subsequently, we applied the longitudinal axis segmentation method described by Morais-Ribeiro et al. [13] to divide the hippocampus into three equal subdivisions: anterior, intermediate, and posterior. Controlling for age, sex, and eTIV, ADc participants exhibited decreased volumes in all three subregions compared with the other groups, in both hemispheres (*P* < 0.001; Fig. 2A, Online supplemental data - Fig S4A). Furthermore, adjusting for eTIV and sex, a similar pattern of correlation with age was observed for the subregional volumes on both hemispheres (*P* < 0.001). Compared with biomarker-negative participants, the ADc group showed weaker age-volume associations in the right posterior and intermediate hippocampus, as well as in the left posterior hippocampus (*P* < 0.025). When compared with SNAP, weaker age-volume associations in ADc were observed across multiple right hippocampal subregions and in the left posterior and intermediate hippocampus (*P* < 0.025) (Fig. 2B, Online supplemental data - Fig S4B).

**Fig. 3:**
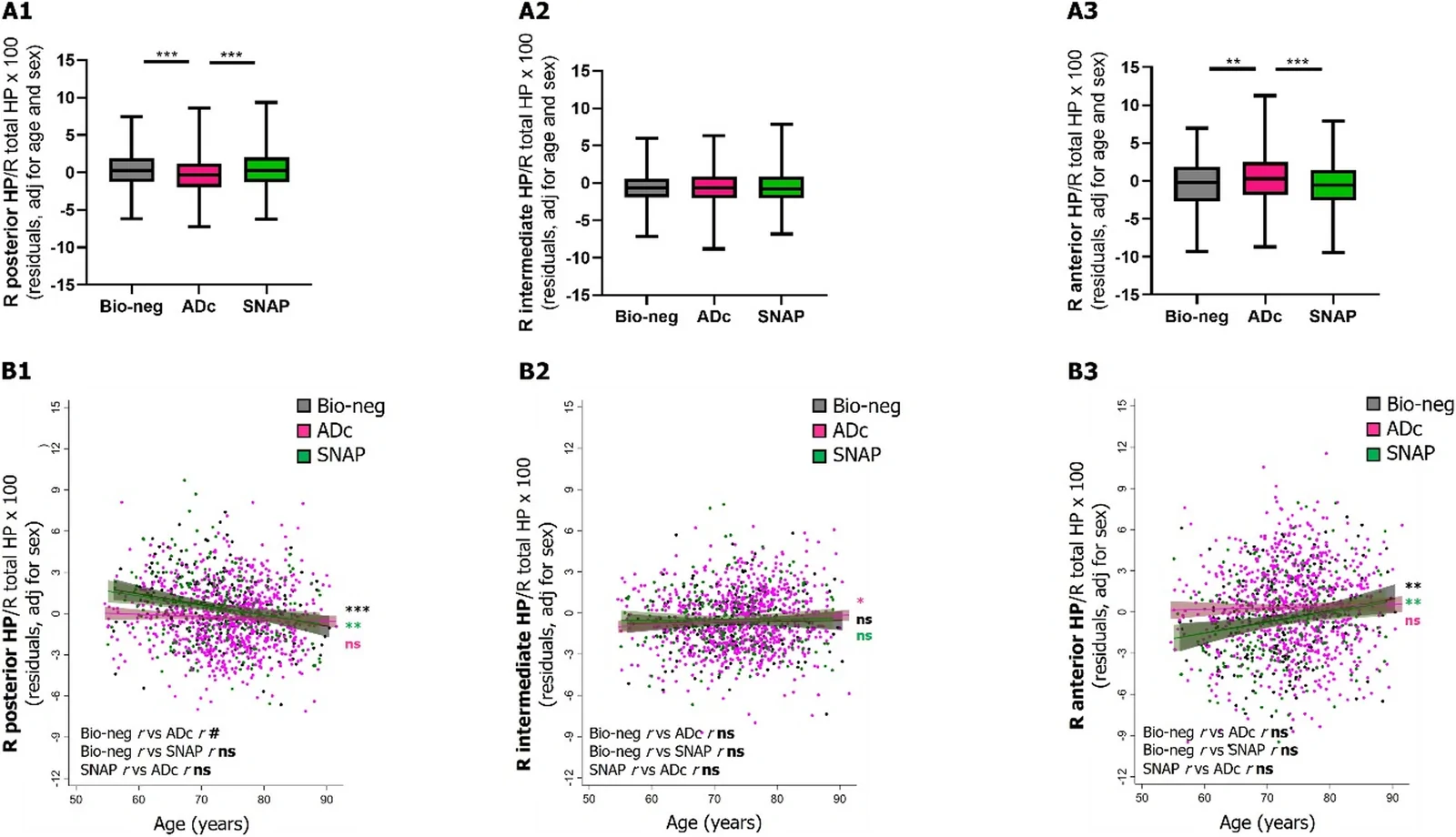
Comparisons between groups of subregional hippocampal volumes normalized for ipsilateral total hippocampal volume on the right hemisphere. Controlling for total right hippocampal (HP) volume, age and sex, ADc has reduced posterior (**A1**) and greater anterior volumes (**A3**) compared to the other groups; no differences are seen in intermediate HP (**A2**). Over age, adjusting for sex and right total HP volume, there is significant decrease of the right posterior HP in biomarker-negative participants and SNAP, with no significant change in ADc (**B1**); ADc has a significant increase in the intermediate HP volume ratio by ipsilateral total HP volume over age, whereas no significant correlations are revealed in the other groups (**B2**); anterior HP volume has a positive significant correlation with age in biomarker-negative participants and SNAP, with no significant correlation identified in ADc (**B3**) – ADc has a significantly weaker correlation compared to biomarker-negative participants for posterior right HP, with no other differences found between the remaining correlations. Detailed statistical results are provided in Online supplemental data. # *P* < 0.01(6); *P < 0.05; **P < 0.01; ***P < 0.001; bio-neg, biomarker-negative participants; ns, non-significant; R, right

To investigate differential intrahippocampal changes in these subregional volumes, we normalized the volumes of the anterior, intermediate, and posterior hippocampus to the total ipsilateral hippocampal volume (besides adjustments for age and sex). ADc participants exhibited an increased ratio of anterior hippocampus volume to total hippocampal volume and a decreased ratio of posterior hippocampus volume to total hippocampal volume compared with biomarker-negative participants or SNAP, both on the left (*P* < 0.001) and right (*P* < 0.01) hemispheres (Fig. 3A, Online supplemental data – Fig S5A). Notably, ADc showed minimal change in these ratios over time, as opposed to biomarker-negative participants or SNAP (exception made for left anterior hippocampus in biomarker-negative participants), in which those ratios tend to equalize the ones from ADc in older individuals (Fig. 3B, Online supplemental data - Fig S5B). No differences between groups were found in intermediate hippocampus volume, including over time.

**Fig. 4:**
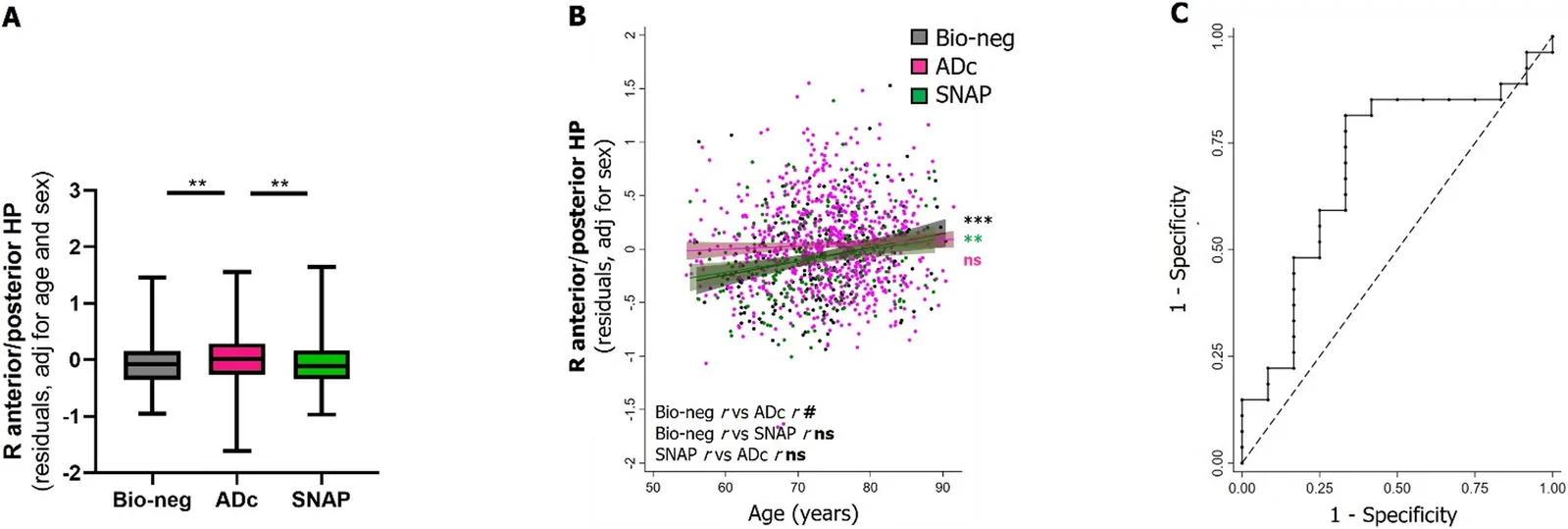
Anterior/posterior right hippocampal volume ratio compared between groups. On the right hippocampus (HP), ADc also display increased anterior/posterior HP volume ratio compared to the other groups (**A**). Over age and controlling for sex, there is significant increase of this ratio in biomarker-negative and SNAP, with no significant change in ADc (**B**) – that variation is significantly different between biomarker-negative participants and ADc. AUC of ROC for the right ratio to distinguish ADc from SNAP in individuals younger than 60 years is estimated at 0.70 (**C**). Detailed statistical results are provided in Online supplemental data. **#** *P* < 0.01(6); **P < 0.01; ***P < 0.001; bio-neg, biomarker-negative participants; ns, non-significant; R, right

To consolidate these differential changes along the longitudinal axis of the hippocampus into a single metric, we calculated the ratio of anterior hippocampal volume to posterior hippocampal volume for both hemispheres. This ratio was bilaterally increased in ADc compared with the other groups (*P* < 0.001; Fig. 4A, Online supplemental data - Fig S6A), and while SNAP and biomarker-negative participants showed progressive increase over time (*P* < 0.01), ADc exhibited minimal changes (Fig. 4B, Online supplemental data - Fig S6B). Given that these volumetric differences between groups attenuate over time and the challenge to distinguish neurodegenerative conditions in younger individuals, we sought to evaluate the potential discriminatory ability of this ratio to correctly discern between SNAP and ADc in young individuals (below 60 years old), by calculating the corresponding AUC of ROC curve for right and left hippocampus. This subgroup comprised 27 ADc participants (17 female, 63.0%) and 12 SNAP participants (8 female, 66.7%). Median age was 56.9 years (interquartile range 2.6) in ADc and 59.0 years (interquartile range 3.3) in SNAP. There were no differences between groups in sex distribution (Fisher’s exact test, *P* = 1.00) or age (Mann–Whitney U test, *P* = 0.13). Clinical status differed between groups in this younger subgroup, with lower MMSE scores in ADc than in SNAP (median 25.0, interquartile range 4.0, *n* = 24 vs. median 29.0, interquartile range 2.0, *n* = 11; Mann–Whitney U test, *P* < 0.001), and higher CDR scores in ADc (median 0.5, interquartile range 0.5 vs. median 0.5, interquartile range 0.0; Mann–Whitney U test, *P* = 0.03). In this younger subgroup, we observed minimally satisfactory discrimination between groups, with an estimated AUC of 0.70 for the right side (Fig. 4C) and 0.65 for the left (Online supplemental data - Fig S6C). When considering the full cohort, discriminatory performance was lower, with AUC values of 0.59 (right) and 0.60 (left).

### Relationship between subregional metrics and clinical scores

**Fig. 5:**
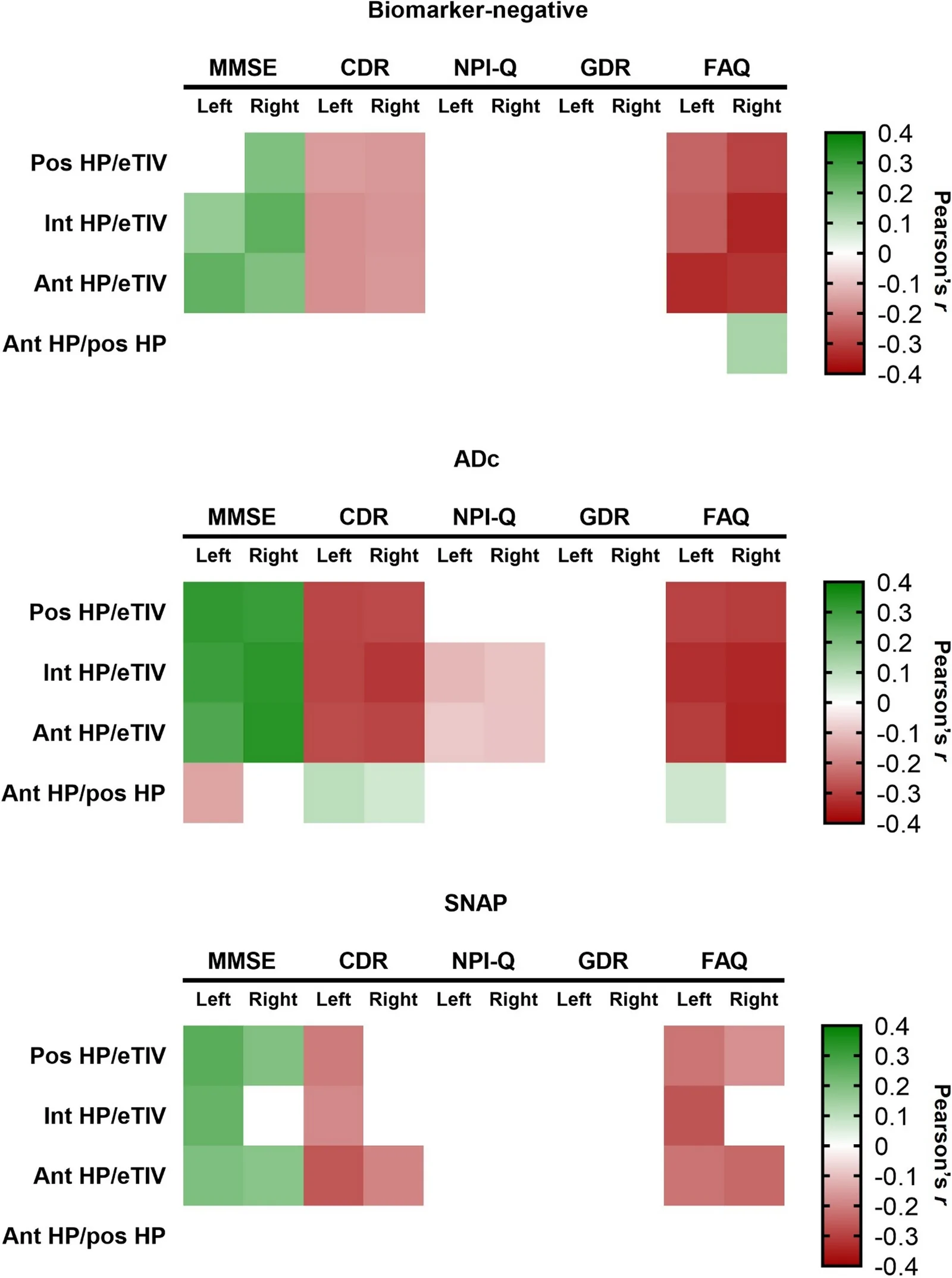
Correlation analyses between hippocampal subregional volumetric measures and various clinical scores. In all of these analyses, adjustment for age and sex was performed. Only significant results are colored, after correction for multiple comparisons with Benjamini-Hochberg method (FDR < 5%). Color and brightness indicate magnitude of Pearson coefficient *r* according to the color code displayed. Biomarker-negative participants, ADc and SNAP show various significant correlations between hippocampal (HP) subregional metrics and mini-mental state exam score, clinical dementia rating and functional activities questionnaire score; no significant correlations were found with geriatric depression scale; ADc is the only group in which there are significant correlations with neuropsychiatric inventory Q score. Refer to Online supplemental data - Tables S1, S2 and S3 for detailed Pearson coefficients, corresponding *P* values and adjusted alpha level that indicates statistical significance. MMSE, Mini-Mental State Exam, CDR, Clinical Dementia Rating; NPI-Q, Neuropsychiatric Inventory Q; GDR, Geriatric Depression Scale; FAQ, Functional Activities Questionnaire

Controlling for age and sex, ADc participants demonstrated worse cognition (*P* < 0.001), more neuropsychiatric symptoms (*P* < 0.01), and greater functional impairment (*P* < 0.001) compared with biomarker-negative participants or SNAP (Online supplemental data - Fig S7A-E). Correlation heatmaps revealed significant associations between hippocampal subregional volumetric measures and clinical scores across groups, with stronger and more widespread correlations in ADc (Fig. 5). In this group, posterior, intermediate, and anterior hippocampal volumes showed bilateral correlations with global cognition (MMSE, *r* values ranging from 0.28 to 0.34), dementia severity (CDR, *r* values ranging from − 0.31 to -0.28) and functional status (FAQ, *r* values ranging from − 0.35 to -0.30). Associations with neuropsychiatric symptoms in ADc were of smaller magnitude (NPI-Q, *r* values ranging from − 0.11 to -0.09) and were restricted to anterior and intermediate hippocampal subregions. In SNAP and biomarker-negative participants, correlations between hippocampal subregional volumes and the clinical scores MMSE and CDR were weaker (MMSE, *r* values below 0.26; CDR, *r* absolute values below 0.26). The ratio of anterior hippocampal volume to posterior hippocampal volume was significantly associated with MMSE, CDR and FAQ in ADc (*r* absolute values ranging from 0.07 to 0.14) and with FAQ in biomarker-negative participants (*r* = 0.13). Notably, more “predictive” variables were detected on the left than the right, for both SNAP and ADc.

To assess the longitudinal clinical relevance of hippocampal subregional volumes, we examined the association between baseline volumetric measures and 2-year change in MMSE and CDR, controlling for age, sex, and baseline performance (Online supplemental data – Table S4). In ADc, lower baseline volume of the right posterior hippocampus was associated with greater cognitive decline over time, reflected by a decrease in MMSE scores (*r* = 0.18) and an increase in CDR scores (*r* = -0.17). In addition, lower baseline volume of the left posterior hippocampus was associated with worsening CDR scores in both ADc (*r* = -0.17) and SNAP (*r* = -0.34) participants. In SNAP, a similar association with CDR progression was also observed for the left intermediate hippocampal volume (*r* = -0.39).

### Association between subregional metrics and CSF biomarkers

**Fig. 6:**
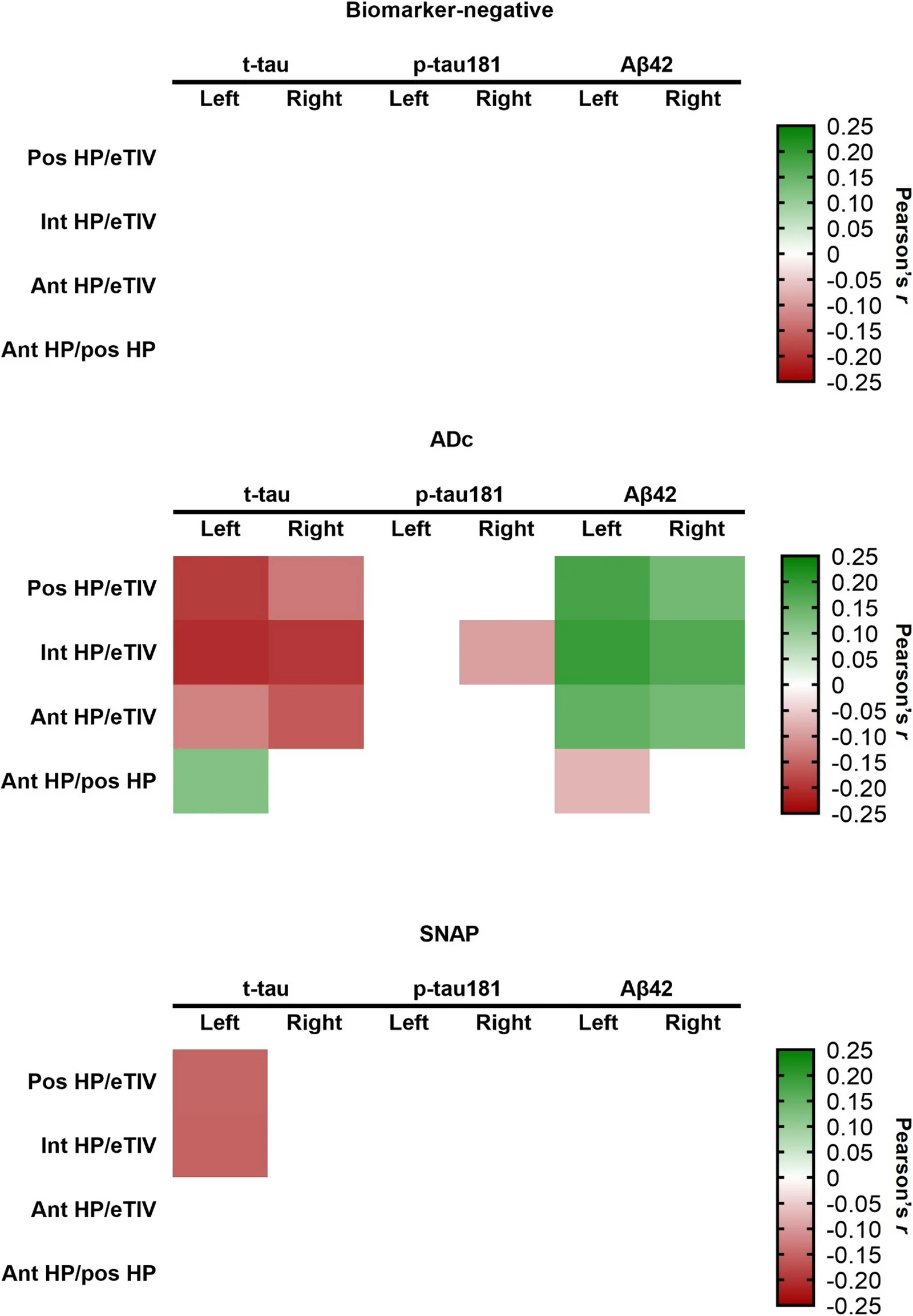
Heat map of correlation analyses (*r*) between hippocampal subregional volumetric measures and CSF levels of t-tau, p-tau181 and Aβ42, for biomarker-negative participants (**A**), ADc (**B**) and SNAP (**C**). Adjustment for age and Benjamini-Hochberg method (FDR < 5%) was used to control for multiple comparisons. Only significant results are colored, according to the color code displayed. Refer to Online supplemental data - Tables S5, S6 and S7 for detailed Pearson coefficients, corresponding *P* values and adjusted alpha level that indicates statistical significance

To depict if these subregional volumes correlate with the CSF biomarkers used to define our groups of study (t-tau, p-tau181 and Aβ42), we built correlation heatmaps between those and the same subregional volume variables. Biomarker-negative participants showed no significant correlations between hippocampal subregional volumes and CSF biomarkers (Fig. 6A). In ADc, hippocampal subregional volumes showed associations with both tau and Aβ pathology (Fig. 6B). Volumes of posterior, intermediate, and anterior hippocampus were bilaterally correlated with CSF t-tau (*r* values ranging from − 0.21 to -0.12) and with CSF Aβ42 levels (*r* values ranging from 0.14 to 0.20). In addition, the anterior-to-posterior hippocampal volume ratio was associated with both t-tau (*r* = 0.12) and Aβ42 (*r* = -0.07) levels in the left hemisphere, and right intermediate hippocampus volume showed an isolated association with p-tau181 (*r* = -0.10) in ADc. In SNAP, significant associations were restricted to CSF t-tau levels, which correlated negatively with posterior and intermediate hippocampal volumes in the left hemisphere (*r* = -0.15) (Fig. 6C).

To further explore the clinical relevance of CSF biomarkers across groups, we examined correlations between CSF t-tau, p-tau181 and Aβ42 levels and clinical scores (Online supplemental data – Tables S8, S9, S10). In biomarker-negative participants, no significant associations were observed between CSF biomarkers and any clinical measures. In contrast, in ADc, CSF t-tau, p-tau181 and Aβ42 levels were significantly associated with global cognition (MMSE), dementia severity (CDR), neuropsychiatric symptoms (NPI-Q), and functional status (FAQ) (*P* ≤ 0.001). In SNAP, significant associations were restricted to CSF t-tau levels, which correlated with MMSE (*r* = − 0.20, *P* = 0.003).

Overall, these results indicate that ADc is characterized by a marked reduction in total and subregional hippocampal volumes compared with biomarker-negative participants and SNAP, with a relatively weaker influence of age on hippocampal volume decline. Along the longitudinal axis, hippocampal atrophy in ADc is disproportionately greater in the posterior than in the anterior subregion, resulting in an increased anterior-to-posterior volume ratio that remains relatively stable with age. Across groups, subregional hippocampal volumetric measures show distinct associations with clinical scales. Finally, subregional hippocampal volumes are associated with CSF biomarkers in a group-specific manner, correlating with both tau and Aβ42 levels in ADc, and with total tau in SNAP.

## Discussion

CSF biomarkers have become integral in aiding the differential diagnosis of neurodegenerative conditions such as ADc, evidenced by Aβ pathology, and SNAP, characterized by elevated tau levels in CSF alongside normal Aβ concentration [3]. Notably, studies focused on the hippocampus have underscored distinct properties along its longitudinal axis [9]. Here, we detail volumetric changes along the hippocampus long axis among individuals with these neurodegenerative conditions and biomarker-negative participants, alongside exploring their relationship with clinical scores and the CSF biomarkers t-tau, p-tau181, and Aβ42.

The hippocampus plays a pivotal role in learning and memory, and its volume is known to decrease in patients with AD [21]. Our subregional analyses align with recent work by Morais-Ribeiro et al. [13], wherein similar investigations were conducted within an AD group defined by clinical presentation. Both studies underscore the heightened vulnerability of the posterior hippocampus to atrophy (Figs. 3 and 4, Online supplemental data – Fig S5). Importantly, interpretation of hippocampal subregional volumes normalized to total hippocampal volume provides insight into relative regional vulnerability. In ADc, the reduced posterior hippocampus-to-total hippocampus ratio indicates a disproportionate loss of posterior hippocampal tissue relative to the overall hippocampal volume. Conversely, the increased anterior hippocampus-to-total hippocampus ratio does not reflect preservation or volumetric increase of the anterior hippocampus, but rather results from greater atrophy affecting other hippocampal segments, particularly the posterior region. Complementing these findings, Wolf et al. employed three-dimensional surface mapping to assess the microstructural integrity of hippocampal subfields, revealing that the association of age with hippocampal microstructural integrity predominantly affects regions situated in the hippocampal body and tail [22]. Moreover, our findings indicate that changes in various volumetric subregions exhibit a less pronounced effect of aging in AD compared with biomarker-negative participants and SNAP. Although ADc participants were older on average, our volume analyses along age indicate that hippocampal atrophy in ADc is already present at younger ages and follows a distinct trajectory, suggesting that disease-related pathological processes outweigh the contribution of chronological aging in shaping hippocampal volumes in this group.

We confirm that volumes of the anterior, intermediate, and posterior hippocampus significantly correlate with MMSE scores [13] and expand upon that by demonstrating that these subregional volumes are also associated with CDR score and daily functioning (Fig. 5). Importantly, beyond cross-sectional associations, our longitudinal analyses indicate that baseline hippocampal subregional volumes - particularly those of the posterior hippocampus - are associated with subsequent cognitive decline over approximately two years. These findings underscore the clinical relevance of posterior hippocampal vulnerability and suggest that posterior hippocampal atrophy is not only a marker of current disease severity but may also reflect future clinical progression. The identification of these associations in groups defined by CSF tau and Aβ biomarkers is consistent with models of AD that propose a temporal cascade whereby Aβ accumulation and tau pathology precede brain atrophy and, ultimately, measurable cognitive decline [23].

Interestingly, our analysis reveals a distinct pattern in ADc, whereby volumes of the intermediate and anterior hippocampus are significantly associated with NPI-Q scores, reflecting the burden of neuropsychiatric symptoms. This finding complements recent research reporting a prevalence of psychosis of approximately 42% in AD, which has been associated with greater disease severity, multi-domain cognitive deficits, heterogeneous neuropathology, and temporal lobe atrophy [24]. Importantly, the functional and clinical relevance of hippocampal subregional alterations is likely to be disease-specific. In schizophrenia, for example, although abnormalities have been reported along the entire longitudinal axis of the hippocampus [25, 26], substantial evidence points to a predominant involvement of anterior hippocampal regions [27]. The anterior hippocampus has been consistently implicated in anxiety-related behaviors [28]; in contrast, volumetric changes associated with depression have more often been reported in posterior hippocampal regions [29, 30]. Taken together, these observations underscore that the interpretation of hippocampal subregional alterations must be contextualized within the specific neuropsychiatric disorder under study. Moreover, given the existence of functionally relevant intrinsic hippocampal circuits spanning the longitudinal axis [31], predominantly posterior atrophy may contribute to functional alterations in anterior hippocampal networks, such that structural vulnerability in one subregion may manifest as functional disturbances in others.

The ratio of anterior hippocampal volume to posterior hippocampal volume, serving as a consolidated metric capturing both anterior and posterior relative atrophy patterns, not only exhibited significant correlations with global cognition and daily functioning in ADc (Fig. 5) but may also provide complementary neuroimaging information to aid in the distinction between ADc and SNAP at younger ages (Fig. 4). Specifically, this ratio showed a minimally satisfactory discriminatory performance when analyses were restricted to participants younger than 60 years of age, whereas discrimination was substantially attenuated when considering the full cohort. This reduction in performance in the full cohort is consistent with the progressive convergence of anterior-posterior hippocampal volumetric patterns across groups with increasing age. In this context, the ratio should not be viewed as a standalone imaging marker applicable across the general population, but rather as a contextual metric whose interpretability depends on the presence of biochemical evidence of neurodegeneration. Accordingly, its potential utility lies in biomarker-informed settings, where it may help refine the differentiation between biologically distinct conditions such as ADc and SNAP that share evidence of tau-related pathology. Given the relatively small sample size of the younger subgroup, these findings should be interpreted as exploratory and warrant replication in independent cohorts.

Our findings regarding subregional volumes in SNAP are consistent with various studies showing that total hippocampal atrophy and cognitive decline are more pronounced in ADc than in SNAP, with the latter showing no relevant differences compared with controls [32–38]. Expanding on those studies focused on total hippocampal volume assessment, our approach delves into anterior-posterior subregional volumes. Interestingly, while SNAP presents as a relatively benign condition at the cognitive and volumetric levels when compared with biomarker-negative participants, we found significant correlations between subregional volumes and CSF t-tau, as well as between t-tau and global cognition (MMSE), in SNAP but not in biomarker-negative participants, indicative of underlying incipient neurodegeneration in the former. Importantly, early neurodegeneration entails synaptic and neuronal loss, with levels of CSF tau (rather than Aβ) being predictive of future cognitive impairments [39]. These volumetric associations were limited to the left posterior and intermediate hippocampal subregions and were of small effect size, consistent with subtle neurodegenerative changes.

Our analyses further revealed various correlations between subregional hippocampal volumes and the levels of biomarkers implicated in the pathophysiology of ADc and SNAP. Significant associations were observed with t-tau, p-tau181, and Aβ42 in ADc, and with t-tau in SNAP (Fig. 6), which were accompanied by corresponding associations between these CSF biomarkers and clinical outcomes within each group. In ADc, these results are consistent with previous findings regarding total hippocampal volume [40]. The positive association observed between hippocampal subregional volumes and CSF Aβ42 levels may primarily reflect the progressive reduction of CSF Aβ42 concentrations with increasing neurodegeneration and atrophy. However, this finding is also compatible with reports suggesting that, particularly at earlier amyloidosis stages, Aβ-related inflammatory processes may be associated with transient regional volume expansion or cortical thickening [41, 42]. Moreover, in AD cases with dementia, postero-medial network regions that coactivate with the posterior hippocampus overlap with areas exhibiting Aβ and tau deposition [12], and biomarker-confirmed AD has been linked to relatively greater posterior hippocampal involvement [11]. On the other hand, other studies indicate that tau pathology emerges earlier in anterior medial temporal regions, including the transentorhinal/entorhinal cortex and the anterior hippocampus, before later intrahippocampal spread. Notably, tau load in anterior structures is robustly associated with distant atrophy of the posterior hippocampus, potentially reflecting the vulnerability of long-range axonal projections connecting the anterior-lateral entorhinal cortex to the posterior hippocampal formation [14]. Thus, disproportionate posterior hippocampal atrophy in ADc is better interpreted as a volumetric pattern associated with disease progression than as evidence that the earliest tau-related changes necessarily originate in the posterior hippocampus. Beyond Aβ- and tau-related mechanisms, additional co-pathologies may also shape longitudinal hippocampal vulnerability in ADc. Interestingly, recent studies of neuropathological and imaging data of AD patients suggest a strong association between antero-medial temporal regions, such as the anterior hippocampus, and the accumulation of TDP-43 [10], a finding replicated in clinically defined populations exhibiting probable TDP-43 pathology [11]. Furthermore, in AD, iron deposition in the posterior hippocampal CA1 region has been strongly and negatively correlated with memory fidelity [43]. Additionally, other pathologies co-occurring in ADc, such as the presence of Lewy bodies [6] or cerebrovascular changes [15], which are known to differentially affect brain atrophy patterns, can potentially impact the hippocampus along its longitudinal axis and should be taken into account in future studies.

On the other hand, cognitive deterioration in SNAP subjects appears to be more closely related to neuronal damage rather than Aβ pathology [44]. In this context, SNAP is conceptually understood as a condition positive for neurodegeneration, which can be defined based on abnormal ¹⁸F-FDG PET scans, MRI-detected atrophy, or elevated CSF tau levels [3]. Despite our findings that SNAP group exhibit increased t-tau levels and corresponding correlations with hippocampal subregional volumes, these volume metrics are not significantly different from biomarker-negative participants. This suggests that different neurodegeneration biomarkers track distinct aspects of disease processes, providing similar but not redundant information [45]. Furthermore, the underlying pathology of SNAP is heterogeneous, and several pathological processes can be encompassed by its definition. These include cerebrovascular disease, α-synucleinopathies, TDP-43 proteinopathy, hippocampal sclerosis, argyrophilic grain disease, traumatic brain injury, corticobasal degeneration, primary progressive aphasia, Creutzfeldt–Jakob disease [7], as well as PART [46, 47]. Notably, PART represents the most prevalent pathology that can be observed in people with “pathological” SNAP (that is, with no evidence of Aβ pathology in the presence of tau pathology) [48, 49]. Additionally, PART typically has a much more limited impact on cognition compared with AD as well, so it may account for at least a subset of individuals with the SNAP biomarker profile [50].

Our analyses revealed that left subregional volumetric metrics are particularly predictive of global cognition and daily functioning in both ADc and SNAP (Fig. 5), the same side where correlations with t-tau were found in SNAP (Fig. 6). Additionally, we observed differences between ADc and SNAP in their correlations of total (Fig. 1, Online supplemental data - Fig S3) and intermediate (Online supplemental data - Fig S3 and S4) hippocampal volumes with age, also on the left side. Altogether, left hippocampal atrophy may better reflect clinical presentation and represent underlying pathophysiology.

Some caveats must be considered in interpreting our findings. First, there may be a degree of selection bias inherent in the ADNI cohort’s composition and corresponding data collection methods. Second, despite the large sample size, relying on a single database may limit the generalizability of our results. Third, despite adjusting for well-established potential confounders, as an observational study, there may be other confounding factors that we did not account for. Importantly, the groups analyzed in this study were defined based on CSF biomarker profiles rather than on detailed pathological confirmation. As discussed above, SNAP represents a heterogeneous biomarker category that may encompass several non-AD pathological processes. In addition, the group defined here as ADc may also be pathologically heterogeneous, as reduced CSF Aβ42 levels have been reported in a range of neurological conditions beyond AD [51]. In particular, PART [49], corticobasal degeneration [52], multiple sclerosis [53, 54], and Creutzfeldt–Jakob disease [55] may present with elevated CSF tau levels and reduced CSF Aβ concentrations. Accordingly, we cannot fully exclude the presence of individuals with these conditions in the SNAP and ADc groups. Such overlap, together with potential longitudinal transitions of individuals of these conditions from a SNAP to an ADc biomarker profile at more advanced disease stages, may have contributed to attenuated group differences between SNAP and biomarker-negative participants. Finally, Aβ pathology in this study was defined using CSF Aβ42 levels alone, rather than ratios such as Aβ42/40 or Aβ42/38, recognized as more robust markers of AD Aβ pathology [56].

As a future perspective, given that AD mainly declarative memory deficits [57], it is crucial to understand the extent to which subregional atrophy patterns along the longitudinal hippocampal axis impact specific components of this form of long-term memory. For instance, in rodents, dorsal hippocampus lesions impair spatial memory and lesions in ventral hippocampus lead to anxiety-like behavior [9, 58], whereas social memory depends on different subregions along the longitudinal axis [31]. Lastly, SNAP’s etiology remains unclear, with several processes possibly contributing to neurodegeneration. Importantly, while the revised criteria for AD diagnosis and staging acknowledge that SNAP cases could correspond to PART, they do not tackle the biomarker definition and potential clinical significance of SNAP and how it differentiates from AD [4]. Therefore, complementary biomarkers reflecting disease-specific and co-occurring pathologies will be invaluable for improving diagnostic accuracy. These biomarkers may encompass genetic, molecular, imaging, and vascular factors [34].

## Conclusions

We demonstrated that ADc displays lower total and subregional hippocampal volumes compared with SNAP or biomarker-negative participants, with a particular susceptibility in the posterior hippocampus and less dependency of these volumetric measures on age. Several subregional hippocampal volumes correlate significantly with altered levels of CSF biomarkers in both ADc and SNAP, aligning with the specific pathophysiology involved, and are associated with global cognition, neuropsychiatric features, and functional impairment. The anterior-posterior relative atrophy pattern may provide complementary information to aid differentiation between ADc and SNAP in biomarker-informed contexts. Therefore, our work contributes to the growing evidence implicating anterior–posterior gradients in hippocampal disease.

## Supplementary Information

Below is the link to the electronic supplementary material.

Supplementary Figure 1

High Resolution Image

Supplementary Figure 2

High Resolution Image

Supplementary Figure 3

High Resolution Image

Supplementary Figure 4

High Resolution Image

Supplementary Figure 5

High Resolution Image

Supplementary Figure 6

High Resolution Image

Supplementary Figure 7

High Resolution Image

Supplementary Material 8

## Data Availability

All data supporting the findings of this manuscript are contained within the manuscript. Additional data are available from the corresponding author upon reasonable request.

## Abbreviations

Aβ: amyloid-β
AD: Alzheimer’s Disease
ADc: Alzheimer’s Disease continuum
ADNI: Alzheimer’s Disease Neuroimaging Initiative
AUC: area under curve
CDR: clinical dementia rating
CSF: cerebrospinal fluid
eTIV: estimated total intracranial volume
FAQ: Functional Activities Questionnaire
GDR: geriatric depression scale
MMSE: mini-mental state exam
MRI: magnetic resonance imaging
NPI-Q: Neuropsychiatric Inventory Q
p-tau181: phosphorylated tau at threonine 181
PART: primary age-related tauopathy
ROC: receiver operating characteristic
SNAP: Suspected Non-AD Pathophysiology
TDP-43: transactive response DNA-binding protein 43
t-tau: total tau
