# Supplementary material for: Differential atrophy along the longitudinal axis of the hippocampus in Alzheimer’s disease and suspected non-Alzheimer’s disease pathophysiology (SNAP)
Source: Neuroradiology. 2026 Jun 6;68(9):2947–61. doi: 10.1007/s00234-026-04061-1 (PMC13680517; doi:10.1007/s00234-026-04061-1)
Supplement: Supplementary file 1 [file 234_2026_4061_MOESM8_ESM.docx]

**Table S1** Correlation analyses between hippocampal subregional volumetric measures and clinical scores in biomarker-negative participants

| **Biomarker-negative participants** | | | | | | | | | | |
| --- | --- | --- | --- | --- | --- | --- | --- | --- | --- | --- |
|  | **MMSE** | | **CDR** | | **NPI-Q** | | **GDS** | | **FAQ** | |
|  | **Left** | **Right** | **Left** | **Right** | **Left** | **Right** | **Left** | **Right** | **Left** | **Right** |
|  | *r*(df), *P* | *r*(df), *P* | *r*(df), *P* | *r*(df), *P* | *r*(df), *P* | *r*(df), *P* | *r*(df), *P* | *r*(df), *P* | *r*(df), *P* | *r*(df), *P* |
| **Pos HP/eTIV** | 0,11(220),  *P* = 0.09 | 0,20(220),  *P* = 0.003 | -0,16(220),  *P* = 0.02 | -0,16(220),  *P* = 0.02 | -0,15(197),  *P* = 0.03 | -0,15(197),  *P* = 0.13 | -0,08(220),  *P* = 0.23 | -0,10(220),  *P* = 0.15 | -0,24(217),  *P* < 0.001 | -0,30(217),  *P* < 0.001 |
| **Int HP/eTIV** | 0,17(219),  *P* =0.01 | 0,25(217),  *P* < 0.001 | -0,18(219),  *P* = 0.008 | -0,17(217),  *P* = 0.01 | -0,13(196),  *P* = 0.07 | -0,09(195),  *P* = 0.21 | -0,04(219),  *P* = 0.58 | 0,00(217),  *P* = 0.96 | -0,25(216),  *P* < 0.001 | -0,34(214),  *P* < 0.001 |
| **Ant HP/eTIV** | 0,25(220),  *P* < 0.001 | 0,20(220),  *P* = 0.003 | -0,18(220),  *P* = 0.009 | -0,16(220),  *P* = 0.02 | -0,13(197),  *P* = 0.07 | -0,11(197),  *P* = 0.13 | -0,08(220),  *P* = 0.25 | -0,05(220),  *P* = 0.46 | -0,33(217),  *P* < 0.001 | -0,32(217),  *P* < 0.001 |
| **Ant HP/Pos HP** | 0,08(220),  *P* = 0.25 | -0,13(220),  *P* = 0.06 | 0,06(220),  *P* =0.39 | 0,09(220),  *P* = 0.18 | 0,09(197),  *P* = 0.22 | 0,11(197),  *P* = 0.11 | 0,04(220),  *P* = 0.54 | 0,12(220),  *P* = 0.07 | 0,03(217),  *P* = 0.70 | 0,13(217),  *P* = 0.05 |
| **Adj. α** | 0.0250 | 0.0375 | 0.0375 | 0.0375 |  |  |  |  | 0.0375 | 0.0500 |

Last line indicates α level that indicates statistical significance after adjustment for multiple comparisons with Benjamini–Hochberg (FDR < 5%)

**Table S2** Correlation analyses between hippocampal subregional volumetric measures and clinical scores in ADc

| **ADc** | | | | | | | | | | |
| --- | --- | --- | --- | --- | --- | --- | --- | --- | --- | --- |
|  | **MMSE** | | **CDR** | | **NPI-Q** | | **GDS** | | **FAQ** | |
|  | **Left** | **Right** | **Left** | **Right** | **Left** | **Right** | **Left** | **Right** | **Left** | **Right** |
|  | *r*(df), *P* | *r*(df), *P* | *r*(df), *P* | *r*(df), *P* | *r*(df), *P* | *r*(df), *P* | *r*(df), *P* | *r*(df), *P* | *r*(df), *P* | *r*(df), *P* |
| **Pos HP/eTIV** | 0,32(740),  *P* < 0.001 | 0,31(740),  *P* < 0.001 | -0,29(739),  *P* < 0.001 | -0,29(739),  *P* < 0.001 | -0,07(685),  *P* = 0.08 | -0,07(685),  *P* = 0.05 | -0,02(738),  *P* = 0.62 | 0,00(738),  *P* = 0.94 | -0,30(729),  *P* < 0.001 | -0,31(729),  *P* < 0.001 |
| **Int HP/eTIV** | 0,31(740),  *P* < 0.001 | 0,33(738),  *P* < 0.001 | -0,29(739),  *P* < 0.001 | -0,31(737),  *P* < 0.001 | -0,11(685),  *P* = 0.003 | -0,10(683),  *P* = 0.01 | -0,04(738),  *P* = 0.34 | 0,01(736),  *P* = 0.70 | -0,33(729),  *P* < 0.001 | -0,34(727),  *P* < 0.001 |
| **Ant HP/eTIV** | 0,28(741),  *P* < 0.001 | 0,34(740),  *P* < 0.001 | -0,28(740),  *P* < 0.001 | -0,29(739),  *P* < 0.001 | -0,09(686),  *P* = 0.02 | -0,10(685),  *P* = 0.01 | -0,04(739),  *P* = 0.24 | 0,00(738),  *P* = 0.94 | -0,31(730),  *P* < 0.001 | -0,35(729),  *P* < 0.001 |
| **Ant HP/Pos HP** | -0,14(741),  *P* < 0.001 | -0,07(739),  *P* = 0.06 | 0,10(740),  *P* =0.005 | 0,07(738),  *P* = 0.05 | 0,00(686),  *P* = 0.94 | -0.02(684),  *P* = 0.57 | -0,02(739),  *P* = 0.66 | 0,01(737),  *P* = 0.87 | 0,08(730),  *P* = 0.04 | 0,06(728),  *P* = 0.10 |
| **Adj. α** | 0.050 | 0.0375 | 0.050 | 0.050 | 0.0250 | 0.0250 |  |  | 0.050 | 0.0375 |

Last line indicates α level that indicates statistical significance after adjustment for multiple comparisons with Benjamini–Hochberg (FDR < 5%)

**Table S3** Correlation analyses between hippocampal subregional volumetric measures and clinical scores in SNAP

| **SNAP** | | | | | | | | | | |
| --- | --- | --- | --- | --- | --- | --- | --- | --- | --- | --- |
|  | **MMSE** | | **CDR** | | **NPI-Q** | | **GDS** | | **FAQ** | |
|  | **Left** | **Right** | **Left** | **Right** | **Left** | **Right** | **Left** | **Right** | **Left** | **Right** |
|  | *r*(df), *P* | *r*(df), *P* | *r*(df), *P* | *r*(df), *P* | *r*(df), *P* | *r*(df), *P* | *r*(df), *P* | *r*(df), *P* | *r*(df), *P* | *r*(df), *P* |
| **Pos HP/eTIV** | 0,26(211),  *P* < 0.001 | 0,19(211),  *P* = 0.004 | -0,21(211),  *P* = 0.002 | -0,11(211),  *P* = 0.10 | -0,05(175),  *P* = 0.50 | -0,05(175),  *P* = 0.48 | 0,00(211),  *P* = 0.97 | -0,01(211),  *P* = 0.97 | -0,22(211),  *P* < 0.001 | -0,18(211),  *P* = 0.01 |
| **Int HP/eTIV** | 0,24(211),  *P* < 0.001 | 0,11(207),  *P* = 0.11 | -0,19(211),  *P* = 0.006 | -0,13(207),  *P* = 0.07 | -0,07(175),  *P* = 0.37 | -0,08(172),  *P* = 0.31 | -0,05(211),  *P* = 0.47 | 0,05(207),  *P* = 0.49 | -0,27(211),  *P* < 0.001 | -0,14(207),  *P* = 0.04 |
| **Ant HP/eTIV** | 0,20(210),  *P* = 0.003 | 0,19(211),  *P* = 0.007 | -0,26(210),  *P* < 0.001 | -0,19(211),  *P* = 0.005 | -0,04(174),  *P* = 0.60 | -0,06(175),  *P* = 0.42 | 0,03(210),  *P* = 0.70 | 0,00(211),  *P* = 0.97 | -0,22(210),  *P* < 0.001 | -0,24(211),  *P* < 0.001 |
| **Ant HP/Pos HP** | -0,11(209),  *P* = 0.12 | -0,11(210),  *P* = 0.10 | -0,03(209),  *P* =0.68 | 0,002(210),  *P* = 0.97 | 0,04(173),  *P* = 0.56 | 0,05(174),  *P* = 0.49 | 0,02(209),  *P* = 0.77 | -0,03(210),  *P* = 0.65 | 0,01(209),  *P* = 0.87 | 0,06(210),  *P* = 0.39 |
| **Adjusted sig. α** | 0.0375 | 0.0250 | 0.0375 | 0.0125 |  |  |  |  | 0.0375 | 0.0250 |

Last line indicates α level that indicates statistical significance after adjustment for multiple comparisons with Benjamini–Hochberg (FDR < 5%)
**Table S4** Correlation analyses between hippocampal subregional volumetric measures and 2-year change in MMSE and CDR scores

|  | **MMSE** | | | | | | **CDR** | | | | | |
| --- | --- | --- | --- | --- | --- | --- | --- | --- | --- | --- | --- | --- |
|  | **Biomarker-negative** | | **ADc** | | **SNAP** | | **Biomarker-negative** | | **ADc** | | **SNAP** | |
|  | **Left** | **Right** | **Left** | **Right** | **Left** | **Right** | **Left** | **Right** | **Left** | **Right** | **Left** | **Right** |
|  | *r*(df), *P* | *r*(df), *P* | *r*(df), *P* | *r*(df), *P* | *r*(df), *P* | *r*(df), *P* | *r*(df), *P* | *r*(df), *P* | *r*(df), *P* | *r*(df), *P* | *r*(df), *P* | *r*(df), *P* |
| **Pos HP/eTIV** | 0.14(89), *P* = 0.18 | 0.03(89), *P* = 0.76 | 0.14(250), *P* = 0.02 | 0.18(250), *P* = 0.004 | -0.03(42), *P* = 0.87 | 0.07(42), *P* = 0.66 | 0.04(89), *P* = 0.70 | 0.08(89), *P* = 0.48 | -0,17(247),  *P* = 0.006 | -0,17(247),  *P* = 0.007 | -0,34(43),  *P* = 0.02 | -0.33(43),  *P* = 0.03 |
| **Int HP/eTIV** | 0.07(89), *P* = 0.48 | 0.05(89), *P* = 0.62 | 0.11(250), *P* = 0.08 | 0.12(250), *P* = 0.07 | -0.08(42), *P* = 0.63 | 0.04(42), *P* = 0.80 | 0.06(89), *P* = 0.60 | 0.11(89), *P* = 0.31 | -0.06(247), *P* = 0.32 | -0.11(247), *P* = 0.07 | -0.39(43), *P* = 0.008 | -0.34(43), *P* = 0.02 |
| **Ant HP/eTIV** | -0.03(89), *P* = 0.80 | 0.11(89), *P* = 0.32 | 0.09(250), *P* = 0.14 | 0.05(250), *P* = 0.43 | -0.04(41), *P* = 0.80 | 0.10(42), *P* = 0.50 | 0.06(89), *P* = 0.60 | 0.09(89), *P* = 0.37 | -0.09(247), *P* = 0.18 | -0.11(246), *P* = 0.10 | -0.22(42), *P* = 0.16 | -0.22(43), *P* = 0.15 |
| **Ant HP/Pos HP** | -0.20(89), *P* = 0.06 | 0.10(89), *P* = 0.36 | -0.07(250), *P* = 0.24 | -0.12(250), *P* = 0.05 | -0.03(41), *P* = 0.85 | 0.07(41), *P* = 0.66 | 0.00(89), *P* = 0.99 | 0.00(89), *P* = 0.98 | 0.13(247), *P* = 0.07 | 0.06(245), *P* = 0.37 | 0.53(42), *P* = 0.53 | 0.16(42), *P* = 0.16 |
| **Adj. α** |  |  |  | 0.0125 |  |  |  |  | 0.0125 | 0.0125 | 0.025 |  |

Last line indicates α level that indicates statistical significance after adjustment for multiple comparisons with Benjamini–Hochberg (FDR < 5%); gray-shaded cells indicate correlations that remained statistically significant after correction for multiple comparisons

**Table S5** Correlation analyses between hippocampal volumetric measures and CSF t-tau, p-tau181 and Aβ42 in biomarker-negative participants

| **Biomarker-negative participants** | | | | | | | |
| --- | --- | --- | --- | --- | --- | --- | --- |
|  | **t-tau** | | **p-tau181** | | **Aβ42** | |  |
|  | **Left** | **Right** | **Left** | **Right** | **Left** | **Right** |  |
|  | *r*(df), *P* | *r*(df), *P* | *r*(df), *P* | *r*(df), *P* | *r*(df), *P* | *r*(df), *P* |  |
| **Pos HP/eTIV** | -1,20(232),  *P* = 0.07 | -0,11(231),  *P* = 0.10 | 0,06(232),  *P* = 0.38 | 0,10(231),  *P* = 0.15 | -0,01(232),  *P* = 0.90 | -0,01(231),  *P* = 0.89 |  |
| **Int HP/eTIV** | -0,07(230),  *P* = 0.27 | -0,12(229),  *P* = 0.08 | 0,10(230),  *P* = 0.15 | 0,08(229),  *P* = 0.22 | -0,01(230),  *P* = 0.86 | -0,09(229),  *P* = 0.20 |  |
| **Ant HP/eTIV** | -0,05(232),  *P* = 0.42 | -0,12(232),  *P* = 0.06 | 0,10(232),  *P* = 0.12 | 0,04(232),  *P* = 0.50 | 0,01(232),  *P* = 0.88 | -0,06(232),  *P* = 0.34 |  |
| **Ant HP/Pos HP** | 0,06(232),  *P* = 0.37 | 0,02(232),  *P* = 0.72 | 0,04(232),  *P* =0.51 | -0,06(232),  *P* = 0.40 | 0,00(232),  *P* = 1.00 | -0,01(232),  *P* = 0.84 |  |
| **Adjusted sig. α** |  |  |  |  |  |  |  |

Last line indicates α level that indicates statistical significance after adjustment for multiple comparisons with Benjamini–Hochberg (FDR < 5%) **Table S6** Correlation analyses between hippocampal volumetric measures and CSF t-tau, p-tau181 and Aβ42 in ADc

| **ADc** | | | | | | | |
| --- | --- | --- | --- | --- | --- | --- | --- |
|  | **t-tau** | | **p-tau181** | | **Aβ42** | |  |
|  | **Left** | **Right** | **Left** | **Right** | **Left** | **Right** |  |
|  | *r*(df), *P* | *r*(df), *P* | *r*(df), *P* | *r*(df), *P* | *r*(df), *P* | *r*(df), *P* |  |
| **Pos HP/eTIV** | -0,19(766),  *P* = 0.001 | -0,13(766),  *P* < 0.001 | -0,07(781),  *P* = 0.04 | -0,04(781),  *P* = 0.10 | 0,18(781),  *P* < 0.001 | 0,14(781),  *P* < 0.001 |  |
| **Int HP/eTIV** | -0,21(765),  *P* < 0.001 | -0,20(763),  *P* < 0.001 | -0,07(780),  *P* = 0.04 | -0,10(778),  *P* = 0.01 | 0,20(780),  *P* < 0.001 | 0,17(778),  *P* < 0.001 |  |
| **Ant HP/eTIV** | -0,12(767),  *P* < 0.001 | -0,16(766),  *P* < 0.001 | -0,02(782),  *P* = 0.50 | -0,06(781),  *P* = 0.10 | 0,15(782),  *P* < 0.001 | 0,14(781),  *P* < 0.001 |  |
| **Ant HP/Pos HP** | 0,12(767),  *P* = 0.001 | 0,01(765),  *P* = 0.84 | 0,07(782),  *P* =0.06 | 0,00(780),  *P* = 0.98 | -0,07(782),  *P* = 0.04 | -0,03(780),  *P* = 0.35 |  |
| **Adjusted sig. α** | 0.05 | 0.0375 |  | 0.0125 | 0.05 | 0.0375 |  |

Last line indicates α level that indicates statistical significance after adjustment for multiple comparisons with Benjamini–Hochberg (FDR < 5%)

**Table S7** Correlation analyses between hippocampal volumetric measures and CSF t-tau, p-tau181 and Aβ42 in SNAP

| **SNAP** | | | | | | | |
| --- | --- | --- | --- | --- | --- | --- | --- |
|  | **t-tau** | | **p-tau181** | | **Aβ42** | |  |
|  | **Left** | **Right** | **Left** | **Right** | **Left** | **Right** |  |
|  | *r*(df), *P* | *r*(df), *P* | *r*(df), *P* | *r*(df), *P* | *r*(df), *P* | *r*(df), *P* |  |
| **Pos HP/eTIV** | -0,15(220),  *P* = 0.02 | -0,05(220),  *P* = 0.47 | 0,04(222),  *P* = 0.51 | 0,05(222),  *P* = 0.45 | -0,01(222),  *P* < 0.001 | -0,08(222),  *P* < 0.001 |  |
| **Int HP/eTIV** | -0,15(220),  *P* = 0.02 | -0,09(216),  *P* = 0.18 | 0,03(222),  *P* = 0.64 | 0,07(218),  *P* = 0.30 | -0,02(222),  *P* < 0.001 | 0,07(218),  *P* < 0.001 |  |
| **Ant HP/eTIV** | -0,14(218),  *P* = 0.05 | -0,16(220),  *P =* 0.02 | 0,04(220),  *P* = 0.52 | 0,05(222),  *P* = 0.43 | 0,02(220),  *P* < 0.001 | -0,06(222),  *P* < 0.001 |  |
| **Ant HP/Pos HP** | 0,03(218),  *P* = 0.69 | -0.08(220),  *P* = 0.26 | -0,03(220),  *P* =0.70 | -0,04(222),  *P* = 0.53 | 0,02(220),  *P* = 0.04 | -0,13(222),  *P* = 0.35 |  |
| **Adjusted sig. α** | 0.0250 |  |  |  |  |  |  |

Last line indicates α level that indicates statistical significance after adjustment for multiple comparisons with Benjamini–Hochberg (FDR < 5%)

**Table S8** Correlation analyses between CSF t-tau, p-tau181 and Aβ42 and clinical scores in biomarker-negative participants

|  | **Biomarker-negative participants** | | | | |
| --- | --- | --- | --- | --- | --- |
|  | **MMSE** | **CDR** | **NPI-Q** | **GDS** | **FAQ** |
|  | *r*(df), *P* | *r*(df), *P* | *r*(df), *P* | *r*(df), *P* | *r*(df), *P* |
| **t-tau** | -0,06(220), *P* = 0.38 | 0,00(220), *P* = 0.96 | 0,06(197), *P* = 0.381 | -0,04(220), *P* = 0.53 | 0,06(217), *P* = 0.36 |
| **p-tau181** | 0,04(220), *P* = 0.57 | 0,01(220), *P* = 0.86 | 0,07(197), *P* = 0.327 | 0,02(220), *P* = 0.74 | 0,03(217), *P* = 0.68 |
| **Aβ42** | 0,00(220), *P* = 0.97 | 0,02(220), *P* = 0.72 | -0,08(197), *P* = 0.263 | -0,13(220), *P* = 0.06 | 0,01(217), *P* = 0.88 |
| **Adj. α** |  |  |  |  |  |

Last line indicates α level that indicates statistical significance after adjustment for multiple comparisons with Benjamini–Hochberg (FDR < 5%); gray-shaded cells indicate correlations that remained statistically significant after correction for multiple comparisons

**Table S9** Correlation analyses between CSF t-tau, p-tau181 and Aβ42 and clinical scores in ADc

|  | **ADc** | | | | |
| --- | --- | --- | --- | --- | --- |
|  | **MMSE** | **CDR** | **NPI-Q** | **GDS** | **FAQ** |
|  | *r*(df), *P* | *r*(df), *P* | *r*(df), *P* | *r*(df), *P* | *r*(df), *P* |
| **t-tau** | -0,31(727), *P* < 0.001 | 0,24(726), *P* < 0.001 | 0,12(672), *P* = 0.001 | 0,00(725), *P* = 0.911 | 0,24(716), *P* < 0.001 |
| **p-tau181** | -0,22(741), *P* < 0.001 | 0,17(740), *P* < 0.001 | 0,12(686), *P* = 0.001 | 0,02(739), *P* = 0.606 | 0,16(730), *P* < 0.001 |
| **Aβ42** | 0,23(741), *P* < 0.001 | -0,21(740), *P* < 0.001 | -0,15(686), *P* < 0.001 | -0,03(739), *P* = 0.357 | -0,19(730), *P* < 0.001 |
| **Adj. α** | 0.05 | 0.05 | 0.05 |  | 0.05 |

Last line indicates α level that indicates statistical significance after adjustment for multiple comparisons with Benjamini–Hochberg (FDR < 5%); gray-shaded cells indicate correlations that remained statistically significant after correction for multiple comparisons

**Table S10** Correlation analyses between CSF t-tau, p-tau181 and Aβ42 and clinical scores in SNAP

|  | **SNAP** | | | | |
| --- | --- | --- | --- | --- | --- |
|  | **MMSE** | **CDR** | **NPI-Q** | **GDS** | **FAQ** |
|  | *r*(df), *P* | *r*(df), *P* | *r*(df), *P* | *r*(df), *P* | *r*(df), *P* |
| **t-tau** | -0,20(209), *P* = 0.003 | 0,02(209), *P* = 0.76 | 0,01(173), *P* = 0.87 | -0,03(209), *P* = 0.63 | 0,16(209), *P* = 0.017 |
| **p-tau181** | -0,13(211), *P* = 0.07 | 0,04(211), *P* = 0.60 | -0,10(175), *P* = 0.19 | -0,10(211), *P* = 0.16 | 0,03(211), *P* = 0.62 |
| **Aβ42** | -0,02(211), *P* = 0.75 | -0,04(211), *P* = 0.52 | -0,08(175), *P* = 0.26 | -0,07(211), *P* = 0.28 | 0,01(211), *P* = 0.88 |
| **Adj. α** | 0.01(6) |  |  |  |  |

Last line indicates α level that indicates statistical significance after adjustment for multiple comparisons with Benjamini–Hochberg (FDR < 5%); gray-shaded cells indicate correlations that remained statistically significant after correction for multiple comparisons

**Fig. S1** Comparison of CSF Aβ42, t-tau and p-tau181 levels between groups. ADc individuals show reduced Aβ42 levels compared with the other groups; there is no difference between biomarker-negative participants and SNAP in Aβ42 (A) (F(2,1239) = 2210.50, η² = 0.78, *P* < 0.001, ANOVA; biomarker-negative vs ADc *P* < 0.001, biomarker-negative vs SNAP *P* = 0.46, ADc vs SNAP *P* < 0.001, Tukey’s multiple comparisons test). The three groups differ significantly from each other in both t-tau (B) (F(2,1222) = 154.22, η² = 0.20, *P* < 0.001, ANOVA; biomarker-negative vs ADc *P* < 0.001, biomarker-negative vs SNAP *P* < 0.001, ADc vs SNAP *P* < 0.001, Tukey’s multiple comparisons test) and p-tau181 (C) (F(2,1239) = 185.38, η² = 0.23, *P* < 0.001, ANOVA; biomarker-negative vs ADc *P* < 0.001, biomarker-negative vs SNAP *P* < 0.001, ADc vs SNAP *P* < 0.001, Tukey’s multiple comparisons test). ****P* < 0.001; bio-neg, biomarker-negative participants

**Fig. S2** Age and sex proportion characterization. ADc has increased age compared to SNAP; no other age difference is found between groups (A) (F(2,1239) = 6.98, η² = 0.01, *P* < 0.001, ANOVA; biomarker-negative vs ADc *P* < 0.08, biomarker-negative vs SNAP *P* < 0.58, ADc vs SNAP *P* = 0.002, Tukey’s multiple comparisons test). No differences were found in sex proportions between groups (B) (*X*^2^(2,N = 1242) = 1.31, *P* = 0.52. ***P* < 0.01; bio-neg, biomarker-negative participants

**Fig. S3** Comparison of total left hippocampus volume between groups. Controlling for age, sex and eTIV, ADc show significantly diminished volumes of the left hippocampus (HP) compared to biomarker-negative participants and SNAP (A) (F(2,1239) = 47.34, η² = 0.07, *P* < 0.001, ANOVA; biomarker-negative vs ADc *P* < 0.001, biomarker-negative vs SNAP *P* = 0.81, ADc vs SNAP *P* < 0.001, Tukey’s multiple comparisons test). Adjusting for sex and eTIV, the three groups show a significant decrease of the left HP volume with age (B) (biomarker-negative *r*(232) = -0.43, *P* < 0.001; ADc *r*(782) = -0.30, *P* < 0.001; SNAP *r*(222) = -0.48, *P* < 0.001) - ADc correlation is significantly different from SNAP (biomarker-negative vs ADc *X*^2^(1,N = 1018) = 4.27, *P* = 0.04; biomarker-negative vs SNAP *X*^2^(1,N = 458) = 0.41, *P* = 0.52; ADc vs SNAP *X*^2^(1,N = 1008) = 7.58, *P* = 0.006, Jennrich test). **#** *P* < 0.01(6); ****P* < 0.001; bio-neg, biomarker-negative participants; ns, non-significant; L, left

**Fig. S4** Subregional left hippocampal volumes normalized to eTIV comparisons between groups. On the left hemisphere, controlling for eTIV, age and sex, ADc show decreased posterior (A1) (F(2,1238) = 52.98, η² = 0.08, *P* < 0.001, ANOVA; biomarker-negative vs ADc *P* < 0.001, biomarker-negative vs SNAP *P* = 0.98, ADc vs SNAP *P* < 0.001, Tukey’s multiple comparisons test), intermediate (A2) (F(2,1235) = 36.37, η² = 0.06, *P* < 0.001, ANOVA; biomarker-negative vs ADc *P* < 0.001, biomarker-negative vs SNAP *P* = 0.82, ADc vs SNAP *P* < 0.001, Tukey’s multiple comparisons test) and anterior volumes (A3) ((F(2,1237) = 21.13, η² = 0.03, *P* < 0.001, ANOVA; biomarker-negative vs ADc *P* < 0.001, biomarker-negative vs SNAP *P* = 0.57, ADc vs SNAP *P* < 0.001, Tukey’s multiple comparisons test) compared to the other groups. Over age, adjusting for sex and eTIV, the three groups reveal a significant decrease of the left posterior (B1) (biomarker-negative *r*(232) = -0.41, *P* < 0.001; ADc *r*(781) = -0.24, *P* < 0.001; SNAP *r*(222) = -0.46, *P* < 0.001), intermediate (B2) (biomarker-negative *r*(231) = -0.33, *P* < 0.001; ADc *r*(780) = -0.23, *P* < 0.001; SNAP *r*(221) = -0.41, *P* < 0.001), and anterior hippocampal (HP) volume (B3) (biomarker-negative *r*(232) = -0.36, *P* < 0.001; ADc *r*(782) = -0.27, *P* < 0.001; SNAP *r*(220) = -0.40, *P* < 0.001). ADc correlation with age is significantly different from the remaining groups in posterior HP volume (biomarker-negative vs ADc *X*^2^(1,N = 1017) = 6.56, *P* = 0.01; biomarker-negative vs SNAP *X*^2^(1,N = 455) = 0.41, *P* = 0.52; ADc vs SNAP *X*^2^(1,N = 1005) = 10.43, *P* = 0.001, Jennrich test) and from SNAP in intermediate HP (biomarker-negative vs ADc *X*^2^(1,N = 1015) = 1.91, *P* = 0.17; biomarker-negative vs SNAP *X*^2^(1,N = 456) = 0.99, *P* = 0.32; ADc vs SNAP *X*^2^(1,N = 1005) = 6.34, *P* = 0.01, Jennrich test), with no difference between correlations in anterior HP (biomarker-negative vs ADc *X*^2^(1,N = 1018) = 1.54, *P* = 0.21; biomarker-negative vs SNAP *X*^2^(1,N = 455) = 0.31, *P* = 0.58; ADc vs SNAP *X*^2^(1,N = 1005) = 3.50, *P* = 0.06, Jennrich test). **#** *P* < 0.025; ****P* < 0.001; bio-neg, biomarker-negative participants; ns, non-significant; L, left

**Fig. S5** Comparisons between groups of subregional hippocampal volumes normalized for ipsilateral total hippocampal volume on the left hemisphere. Controlling for total left hippocampal (HP) volume, age and sex, ADc show decreased posterior (A1) (F(2,1235) = 17.86, η² = 0.03, *P* < 0.001, ANOVA; biomarker-negative vs ADc *P* < 0.001, biomarker-negative vs SNAP *P* = 0.94, ADc vs SNAP *P* < 0.001, Tukey’s multiple comparisons test) and increased anterior volumes (A3) ((F(2,1237) = 16.62, η² = 0.03, *P* < 0.001, ANOVA; biomarker-negative vs ADc *P* < 0.001, biomarker-negative vs SNAP *P* = 0.75, ADc vs SNAP *P* < 0.001, Tukey’s multiple comparisons test) compared to the other groups, with no difference in intermediate HP (A2) ((F(2,1233) = 0.03, η² < 0.01, *P* = 0.97, ANOVA). Over age, adjusting for sex and left total HP volume, there is significant decrease of the left posterior HP in biomarker-negative participants and SNAP, with no significant change in ADc (B1) (biomarker-negative *r*(232) = -0.21, *P* < 0.001; ADc *r*(779) = -0.04, *P* = 0.28; SNAP *r*(222) = -0.16, *P* = 0.01); no significant changes of intermediate HP volume are seen in any group (B2) (biomarker-negative *r*(230) = 0.11, *P* = 0.09; ADc *r*(779) = 0.07, *P* = 0.06; SNAP *r*(221) = 0.02, *P* = 0.72); anterior HP volume has a positive significant correlation with age in SNAP, with no significant correlation identified in the other groups (B3) (biomarker-negative *r*(232) = 0.10, *P* = 0.12; ADc *r*(782) = -0.01, *P* = 0.84; SNAP *r*(220) = 0.14, *P* = 0.03) – there are no significant differences between groups’s correlations for these subregions (Posterior HP: biomarker-negative vs ADc *X*^2^(1,N = 1015) = 5.56, *P* = 0.01; biomarker-negative vs SNAP *X*^2^(1,N = 458) = 0.30, *P* = 0.58; ADc vs SNAP *X*^2^(1,N = 1005) = 2.76, *P* = 0.10; Intermediate HP: biomarker-negative vs ADc *X*^2^(1,N = 1013) = 0.40, *P* = 0.53; biomarker-negative vs SNAP *X*^2^(1,N = 453) = 0.92, *P* = 0.34; ADc vs SNAP *X*^2^(1,N = 1004) = 0.32, *P* = 0.57; Anterior HP: biomarker-negative vs ADc *X*^2^(1,N = 1018) = 2.16, *P* = 0.14; biomarker-negative vs SNAP *X*^2^(1,N = 456) = 0.18, *P* = 0.67; ADc vs SNAP *X*^2^(1,N = 1006) = 3.84, *P* = 0.05, Jennrich test). **P* < 0.05; ***P* < 0.01; ****P* < 0.001; bio-neg, biomarker-negative participants; ns, non-significant; L, left

**Fig. S6** Anterior/posterior left hippocampal volume ratio compared between groups. Adjusting for total left hippocampal (HP) volume, age and sex, ADc show increased ratio of anterior HP volume divided by posterior HP volume on the left (A) (F(2,1237) = 20.03, η² = 0.03, *P* < 0.001, ANOVA; biomarker-negative vs ADc *P* < 0.001, biomarker-negative vs SNAP *P* > 0.99, ADc vs SNAP *P* < 0.001, Tukey’s multiple comparisons test). With aging, controlling for sex, there is significant increase of this ratio in biomarker-negative participants and SNAP, with no significant change in ADc (B) (biomarker-negative *r*(232) = 0.19, *P* = 0.004; ADc *r*(782) = 0.03, *P* = 0.48; SNAP *r*(220) = 0.18, *P* = 0.009) – there are no significant differences between groups’s correlations on the left (biomarker-negative vs ADc *X*^2^(1,N = 1018) = 4.88, *P* = 0.03; biomarker-negative vs SNAP *X*^2^(1,N = 456) = 0.02, *P* = 0.88; ADc vs SNAP *X*^2^(1,N = 1006) = 3.92, *P* = 0.05, Jennrich test). AUC of ROC using left anterior HP volume/posterior HP volume as predictor of ADc versus SNAP in individuals younger than 60 years is estimated at 0.65 (C). ***P* < 0.01; ****P* < 0.001; bio-neg, biomarker-negative participants; ns, non-significant; L, left

**Fig. S7** Comparison between groups for various clinical scores. In all of these analyses, adjustment for age and sex was performed. ADc participants show decreased Mini-Mental State Exam scores compared with biomarker-negative participants and SNAP (A) (F(2,1175) = 82.28, η² = 0.12, *P* < 0.001, ANOVA; biomarker-negative vs ADc *P* < 0.001, biomarker-negative vs SNAP *P* = 0.86, ADc vs SNAP *P* < 0.001, Tukey’s multiple comparisons test). Clinical Dementia Rating scores are increased in ADc compared with both biomarker-negative participants and SNAP (B) (F(2,1174) = 70.52, η² = 0.11, *P* < 0.001, ANOVA; biomarker-negative vs ADc *P* < 0.001, biomarker-negative vs SNAP *P* = 0.70, ADc vs SNAP *P* < 0.001, Tukey’s multiple comparisons test). Neuropsychiatric Inventory Q scores are higher in ADc compared with biomarker-negative participants and SNAP (C) (F(2,1061) = 15.39, η² = 0.03, *P* < 0.001, ANOVA; biomarker-negative vs ADc *P* = 0.001, biomarker-negative vs SNAP *P* = 0.39, ADc vs SNAP *P* < 0.001, Tukey’s multiple comparisons test). No significant differences between groups are observed for the Geriatric Depression Scale (D) (F(2,1173) = 3.83, η² = 0.01, *P* = 0.02, ANOVA; biomarker-negative vs ADc *P* = 0.11, biomarker-negative vs SNAP *P* = 0.96, ADc vs SNAP *P* = 0.06, Tukey’s multiple comparisons test). Functional Activities Questionnaire scores are increased in ADc compared with biomarker-negative participants and SNAP (E) (F(2,1161) = 69.75, η² = 0.11, *P* < 0.001, ANOVA; biomarker-negative vs ADc *P* < 0.001, biomarker-negative vs SNAP *P* = 0.94, ADc vs SNAP *P* < 0.001, Tukey’s multiple comparisons test). ***P* < 0.01, ****P* < 0.001; bio-neg, biomarker-negative participants; ns, non-significant

**FIGURE LEGENDS (with complete statistical information)**

**Fig. 1** Comparison of total right hippocampus volume between groups. Controlling for age, sex and eTIV, ADc show significantly diminished volumes of the right hippocampus (HP) compared to biomarker-negative participants and SNAP (A) (F(2,1238) = 46.70, η² = 0.07, *P* < 0.001, ANOVA; biomarker-negative vs ADc *P* < 0.001, biomarker-negative vs SNAP *P* = 0.38, ADc vs SNAP *P* < 0.001, Tukey’s multiple comparisons test). Adjusting for sex and eTIV, the three groups show a significant decrease of the right HP volume with age (B) (biomarker-negative *r*(232) = -0.47, *P* < 0.001; ADc *r*(781) = -0.26, *P* < 0.001; SNAP *r*(222) = -0.54, *P* < 0.001) - ADc correlation is significantly different from the other groups (biomarker-negative vs ADc *X*^2^(1,N = 1017) = 9.23, *P* = 0.002; biomarker-negative vs SNAP *X*^2^(1,N = 458) = 1.05, *P* = 0.30; ADc vs SNAP *X*^2^(1,N = 1007) = 16.61, *P* < 0.001, Jennrich test). **#** *P* < 0.01(6); ****P* < 0.001; bio-neg, biomarker-negative participants; ns, non-significant; R, right

**Fig. 2** Subregional right hippocampal volumes normalized to eTIV comparisons between groups. On the right hemisphere, ADc displays reduced volume of posterior (A1) (F(2,1237) = 43.78, η² = 0.07, *P* < 0.001, ANOVA; biomarker-negative vs ADc *P* < 0.001, biomarker-negative vs SNAP *P* = 0.52, ADc vs SNAP *P* < 0.001, Tukey’s multiple comparisons test), intermediate (A2) (F(2,1228) = 32.98, η² = 0.05, *P* < 0.001, ANOVA; biomarker-negative vs ADc *P* < 0.001, biomarker-negative vs SNAP *P* = 0.23, ADc vs SNAP *P* < 0.001, Tukey’s multiple comparisons test) and anterior hippocampus (HP) (A3) (F(2,1238) = 27.71, η² = 0.04, *P* < 0.001, ANOVA; biomarker-negative vs ADc *P* < 0.001, biomarker-negative vs SNAP *P* = 0.47, ADc vs SNAP *P* < 0.001, Tukey’s multiple comparisons test)). The three groups have a significant decrease of the right posterior (B1) (biomarker-negative *r*(232) = -0.46, *P* < 0.001; ADc *r*(781) = -0.22, *P* < 0.001; SNAP *r*(222) = -0.50, *P* < 0.001), intermediate (B2) (biomarker-negative *r*(229) = -0.40, *P* < 0.001; ADc *r*(778) = -0.21, *P* < 0.001; SNAP *r*(218) = -0.47, *P* < 0.001), and anterior HP volume (B3) (biomarker-negative *r*(232) = -0.37, *P* < 0.001; ADc *r*(781) = -0.23, *P* < 0.001; SNAP *r*(222) = -0.44, *P* < 0.001). ADc correlation with age is significantly different from the remaining groups in posterior HP volume (biomarker-negative vs ADc *X*^2^(1,N = 1017) = 11.22, *P* < 0.001; biomarker-negative vs SNAP *X*^2^(1,N = 458) = 0.31, *P* = 0.57; ADc vs SNAP *X*^2^(1,N = 1007) = 15.15, *P* < 0.001, Jennrich test) and in intermediate HP (biomarker-negative vs ADc *X*^2^(1,N = 1011) = 7.25, *P* = 0.007; biomarker-negative vs SNAP *X*^2^(1,N = 451) = 0.89, *P* = 0.34; ADc vs SNAP *X*^2^(1,N = 1000) = 13.58, *P* < 0.001, Jennrich test); in anterior HP, it is different from SNAP (biomarker-negative vs ADc *X*^2^(1,N = 1017) = 4.13, *P* = 0.04; biomarker-negative vs SNAP *X*^2^(1,N = 458) = 0.79, *P* = 0.37; ADc vs SNAP *X*^2^(1,N = 1007) = 9.04, *P* = 0.003, Jennrich test). **#** *P* < 0.025; ****P* < 0.001; bio-neg, biomarker-negative participants; ns, non-significant; R, right

**Fig. 3** Comparisons between groups of subregional hippocampal volumes normalized for ipsilateral total hippocampal volume on the right hemisphere. Controlling for total right hippocampal (HP) volume, age and sex, ADc has reduced posterior (A1) (F(2,1237) = 11.44, η² = 0.02, *P* < 0.001, ANOVA; biomarker-negative vs ADc *P* < 0.001, biomarker-negative vs SNAP *P* > 0.99, ADc vs SNAP *P* < 0.001, Tukey’s multiple comparisons test) and greater anterior volumes (A3) ((F(2,1237) = 9.83, η² = 0.02, *P* < 0.001, ANOVA; biomarker-negative vs ADc *P* = 0.003, biomarker-negative vs SNAP *P* = 0.93, ADc vs SNAP *P* < 0.001, Tukey’s multiple comparisons test) compared to the other groups; no differences are seen in intermediate HP (A2) ((F(2,1223) = 0.39, η² < 0.01, *P* = 0.68, ANOVA). Over age, adjusting for sex and right total HP volume, there is significant decrease of the right posterior HP in biomarker-negative participants and SNAP, with no significant change in ADc (B1) (biomarker-negative *r*(232) = -0.24, *P* < 0.001; ADc *r*(780) = -0.05, *P* = 0.15; SNAP *r*(222) = -0.21, *P* = 0.02); ADc has a significant increase in the intermediate HP volume ratio by ipsilateral total HP volume over age, whereas no significant correlations are revealed in the other groups (B2) (biomarker-negative *r*(228) = 0.02, *P* = 0.76; ADc *r*(776) = 0.08, *P* = 0.04; SNAP *r*(216) = 0.02, *P* = 0.82); anterior HP volume has a positive significant correlation with age in biomarker-negative participants and SNAP, with no significant correlation identified in ADc (B3) (biomarker-negative *r*(232) = 0.19, *P* = 0.004; ADc *r*(780) = 0.03, *P* = 0.47; SNAP *r*(222) = 0.19, *P* = 0.005) – ADc has a significantly weaker correlation compared to biomarker-negative participants for posterior right HP, with no other differences found between the remaining correlations (Posterior HP: biomarker-negative vs ADc *X*^2^(1,N = 1016) = 6.24, *P* = 0.01; biomarker-negative vs SNAP *X*^2^(1,N = 458) = 0.11, *P* = 0.74; ADc vs SNAP *X*^2^(1,N = 1006) = 4.24, *P* = 0.04; Intermediate HP: biomarker-negative vs ADc *X*^2^(1,N = 1008) = 0.53, *P* = 0.47; biomarker-negative vs SNAP *X*^2^(1,N = 448) < 0.01, *P* = 0.96; ADc vs SNAP *X*^2^(1,N = 1004) = 0.61, *P* = 0.43; Anterior HP: biomarker-negative vs ADc *X*^2^(1,N = 1016) = 4.72, *P* = 0.03; biomarker-negative vs SNAP *X*^2^(1,N = 458) < 0.01, *P* = 0.99; ADc vs SNAP *X*^2^(1,N = 1006) = 4.64, *P* = 0.03, Jennrich test). # *P* < 0.01(6); **P* < 0.05; ***P* < 0.01; ****P* < 0.001; bio-neg, biomarker-negative participants; ns, non-significant; R, right

**Fig. 4** Anterior/posterior right hippocampal volume ratio compared between groups. On the right hippocampus (HP), ADc also display increased anterior/posterior HP volume ration compared to the other groups (A) (F(2,1237) = 9.84, η² = 0.02, *P* < 0.001, ANOVA; biomarker-negative vs ADc *P* = 0.001, biomarker-negative vs SNAP *P* > 0.99, ADc vs SNAP *P* = 0.002, Tukey’s multiple comparisons test). Over age and controlling for sex, there is significant increase of this ratio in biomarker-negative participants and SNAP, with no significant change in ADc (B) (biomarker-negative *r*(232) = 0.23, *P* < 0.001; ADc *r*(780) = 0.05, *P* = 0.16; SNAP *r*(220) = 0.20, *P* = 0.002) – that variation is significantly different between biomarker-negative participants and ADc (biomarker-negative vs ADc *X*^2^(1,N = 1016) = 5.99, *P* = 0.01; biomarker-negative vs SNAP *X*^2^(1,N = 456) = 0.11, *P* = 0.75; ADc vs SNAP *X*^2^(1,N = 1006) = 4.06, *P* = 0.04, Jennrich test). AUC of ROC for the right ratio to distinguish ADc from SNAP in individuals younger than 60 years is estimated at 0.70 (C). **#** *P* < 0.01(6); ***P* < 0.01; ****P* < 0.001; bio-neg, biomarker-negative participants; ns, non-significant; R, right

**Fig. 5** Correlation analyses between hippocampal subregional volumetric measures and various clinical scores. In all of these analyses, adjustment for age and sex was performed. Only significant results are colored, after correction for multiple comparisons with Benjamini-Hochberg method (FDR < 5%). Color and brightness indicate magnitude of Pearson coefficient *r* according to the color code displayed. Biomarker-negative participants, ADc and SNAP show various significant correlations between hippocampal (HP) subregional metrics and mini-mental state exam score, clinical dementia rating and functional activities questionnaire score; no significant correlations were found with geriatric depression scale; ADc is the only group in which there are significant correlations with neuropsychiatric inventory Q score. Refer to Online supplemental data - Tables S1, S2 and S3 for detailed Pearson coefficients, corresponding *P* values and adjusted alpha level that indicates statistical significance. MMSE, Mini-Mental State Exam, CDR, Clinical Dementia Rating; NPI-Q, Neuropsychiatric Inventory Q; GDR, Geriatric Depression Scale; FAQ, Functional Activities Questionnaire

**Fig. 6** Heat map of correlation analyses (*r*) between hippocampal subregional volumetric measures and CSF levels of t-tau, p-tau181 and Aβ42, for biomarker-negative participants (A), ADc (B) and SNAP (C). Adjustment for age and Benjamini-Hochberg method (FDR < 5%) was used to control for multiple comparisons. Only significant results are colored, according to the color code displayed. Refer to Online supplemental data - Tables S5, S6 and S7 for detailed Pearson coefficients, corresponding *P* values and adjusted alpha level that indicates statistical significance
